# New Diterpenoids and Isocoumarin Derivatives from the Mangrove-Derived Fungus *Hypoxylon* sp.

**DOI:** 10.3390/md19070362

**Published:** 2021-06-24

**Authors:** Bolin Hou, Sushi Liu, Ruiyun Huo, Yueqian Li, Jinwei Ren, Wenzhao Wang, Tao Wei, Xuejun Jiang, Wenbing Yin, Hongwei Liu, Ling Liu, Erwei Li

**Affiliations:** 1 State Key Laboratory of Mycology, Institute of Microbiology, Chinese Academy of Sciences, Beijing 100101, China; houbl@im.ac.cn (B.H.); liuss@im.ac.cn (S.L.); huory@im.ac.cn (R.H.); yqli19@lzu.edu.cn (Y.L.); renjw@im.ac.cn (J.R.); wangwz@im.ac.cn (W.W.); jiangxj@im.ac.cn (X.J.); yinwb@im.ac.cn (W.Y.); liuhw@im.ac.cn (H.L.); 2 Beijing Key Laboratory of Bioactive Substance and Functional Foods, Beijing Union University, Beijing 100191, China; weitao@buu.edu.cn; 3 Institutional Center for Shared Technologies and Facilities, Institute of Microbiology, Chinese Academy of Sciences, Beijing 100101, China

**Keywords:** mangrove-derived fungus, secondary metabolites, absolute configurations, bioactivity

## Abstract

Two new diterpenoids, hypoxyterpoids A (**1**) and B (**2**), and four new isocoumarin derivatives, hypoxymarins A–D (**4**–**7**), together, with seven known metabolites (**3** and **8**–**13**) were obtained from the crude extract of the mangrove-derived fungus *Hypoxylon* sp. The structures of the new compounds were elucidated on the basis of 1- and 2-dimensional (1D/2D) nuclear magnetic resonance (NMR) spectroscopic and mass spectrometric analysis. The absolute configurations of compounds **1**, **2**, **4**, **5,** and **7** were determined by comparison of experimental and calculated electronic circular dichroism (ECD) spectra, and the absolute configurations of C-4′ in **6** and C-9 in **7** were determined by [Rh_2_(OCOCF_3_)_4_]-induced ECD spectra. Compound **1** showed moderate *α*-glucosidase inhibitory activities with IC_50_ values of 741.5 ± 2.83 μM. Compounds **6** and **11** exhibited DPPH scavenging activities with IC_50_ values of 15.36 ± 0.24 and 3.69 ± 0.07 μM, respectively.

## 1. Introduction

Bioactive secondary metabolites have played an important role for drug discovery as lead compounds [1,2]. Fungi especially from unique ecological niches are capable of producing a variety of bioactive compounds [3,4,5]. Mangrove-derived endophytic fungi, as plant mutualists that occur in the tropical and subtropical intertidal estuarine zones, have attracted more attention of natural product chemists due to their production of structurally diverse and biologically active secondary metabolites [6,7,8,9,10,11,12]. *Hypoxylon* species are widespread in terrestrial and marine environments, and chemical investigations of some *Hypoxylon* spp. have afforded a variety of natural products, such as azaphilones, diterpenes, sporothriolide derivatives, and various aromatic compounds, which exhibited antibacterial, antifungal, and cytotoxic activities [13,14,15,16,17,18].

**Figure 1:**
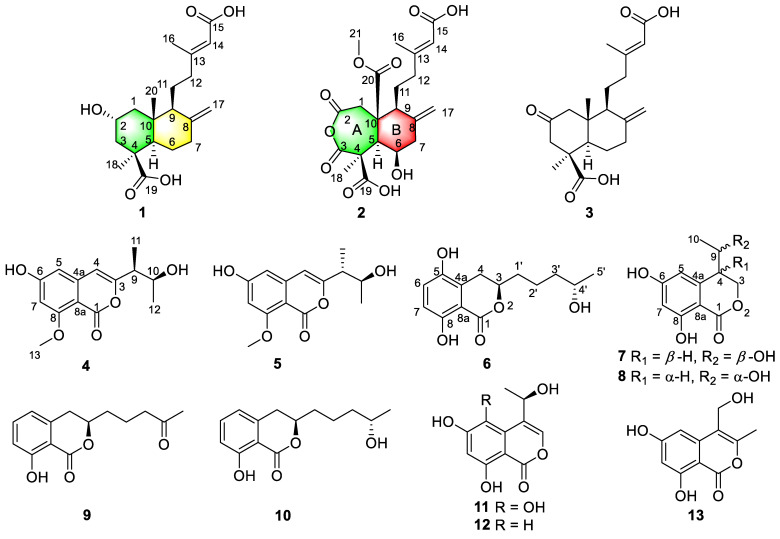
Structures of compounds **1**–**13**.

As part of our ongoing search for new bioactive natural products from mangrove-derived endophytic fungi [11], a strain of fungus *Hypoxylon* sp. (Hsl2-6) from *Bruguiera gymnorrhiza* collected in Fangchenggang City, Guangxi Province, People’s Republic of China, was screened out for investigations. The fungus was fermented on rice medium for 30 days and subsequently extracted by EtOAc to afford the organic extract. Fractionations of this extract were performed, leading to the isolation of two new diterpenoids, hypoxyterpoids A (**1**) and B (**2**), and four new isocoumarin derivatives, hypoxymarins A–D (**4**–**7**), together with seven known metabolites, penicichrysogene B (**3**) [19] penicimarin A (**8**) [20], aspergillumarin A (**9**) [21], aspergillumarin B (**10**) [21], 5-hydroxysescandelin (**11**) [22], sescandelin A (**12**) [23], and sescandelin B (**13**) [24] (Figure 1). Herein, the isolation, structure elucidation and bioactivities of these compounds are reported.

## 2. Results and Discussion

The fungus *Hypoxylon* sp. (Hsl2-6) was cultured in rice medium at room temperature for 30 days. The EtOAc extracts were combined to afford an organic residue (30.0 g) that was fractionated and purified by column chromatography (CC) on silica gel, RP-18, and semipreparative HPLC columns to acquire compounds **1**–**13**.

Hypoxyterpoid A (**1**) was obtained as a brown oil. It had a molecular formula of C_20_H_30_O_5_ (six degrees of unsaturation) on the basis of its high-resolution electrospray ionization mass spectrometry (HRESIMS) [M + Na]^+^ at *m/z* 373.1995 (calcd for C_20_H_30_O_5_Na 373.1991). The infrared (IR) spectrum of **1** at 1704, 1644 cm^−1^ showed the presence of carbonyl and double bond groups. Analysis of the ^1^H and ^13^C nuclear magnetic resonance (NMR, Figures S1 and S2) and heteronuclear single quantum correlations (HSQC) data (Table 1) revealed the presence of two carbonyl carbons (*δ*_C_ 178.3 and 167.6), two sp^3^ quaternary carbons (*δ*_C_ 44.2 and 40.9), four olefinic carbons (two protonated), three sp^3^ methine (one oxygenated), six sp^3^ methylene carbons, and three methyl carbons. These data accounted for all ^1^H and ^13^C NMR resonances except for three unobserved exchangeable protons and suggested that **1** was a bicyclic compound. Analysis of ^1^H-^1^H correlation spectroscopy (COSY) spectrum revealed three spin system: C-1/C-2/C-3, C-5/C-6/C-7, and C-9/C-11/C-12. The heteronuclear multiple bond correlations (HMBC) correlations from H_2_-1 to C-3 and C-5, H_2_-3 to C-5, H-5 to C-4, C-10 and C-18, and from H_3_-18 to C-3, C-4, C-5 and C-19 completed the ring A with the methyl carbon C-18 and the carboxylic carbon C-19 attached to C-4 directly. HMBC correlations from H_3_-20 to C-1, C-5 and C-10 established the location of the methyl group C-20 at C-10. The exchangeable proton was located at C-2 supported by the chemical shift value for C-2 (*δ*_C_ 62.9). While HMBC correlations from H_2_-6 to C-8 and C-10, H-9 to C-8, C-10 and C-20, and from H_2_-17 to C-7, C-8 and C-9 established the ring B, fused with ring A at C-5 and C-10. Additional HMBC correlations from H_3_-16 to C-12, C-13, C-14 and C-15, from H-14 to C-12 and C-15, from H_2_-12 to C-13 and C-14, and from H_2_-11 to C-13 established the C-13–C-16 subunit, with C-12 attached to C-13 of the olefin C-13/C-14. The hydroxyl groups were located at C-2, C-15, and C-19 by default supported by the chemical shift value for C-2 (*δ*_C_ 62.9), C-15 (*δ*_C_ 167.6), and C-19 (*δ*_C_ 178.3). Thus, The compound **1** and *rel*-(1*R*,3*S*,4a*S*,5*R*,8a*S*)-5-[(3*E*)-4-carboxy-3-methylbut-3-en-1-yl]decahydro-3-hydroxy-1,4a-dimethyl-6-methylidenenaphthalene-1-carboxylic [25] shared the same planar structure, as depicted in Figure 1.

**Figure 2:**
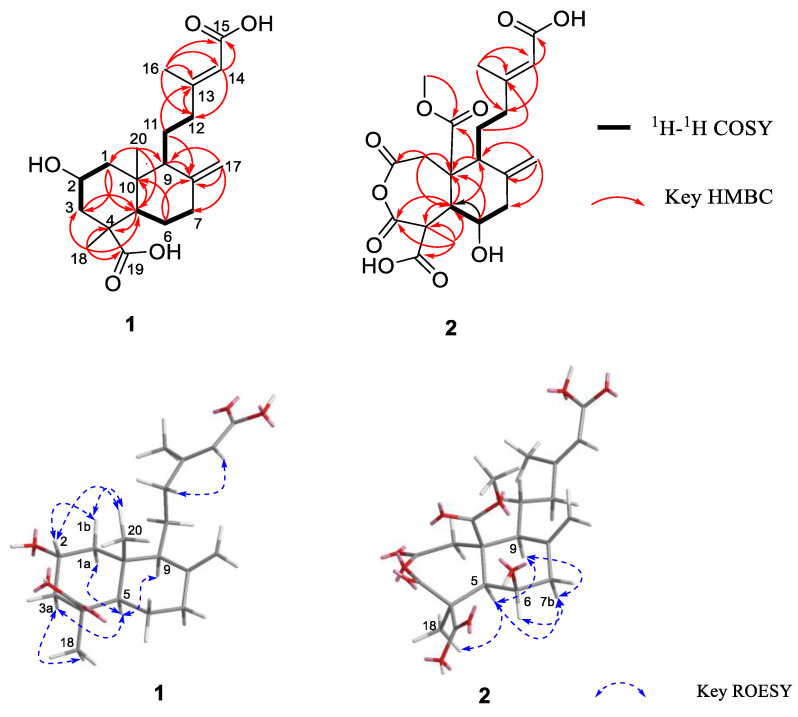
Key ^1^H-^1^H COSY, HMBC and ROESY correlations of **1** and **2**.

**Figure 3:**
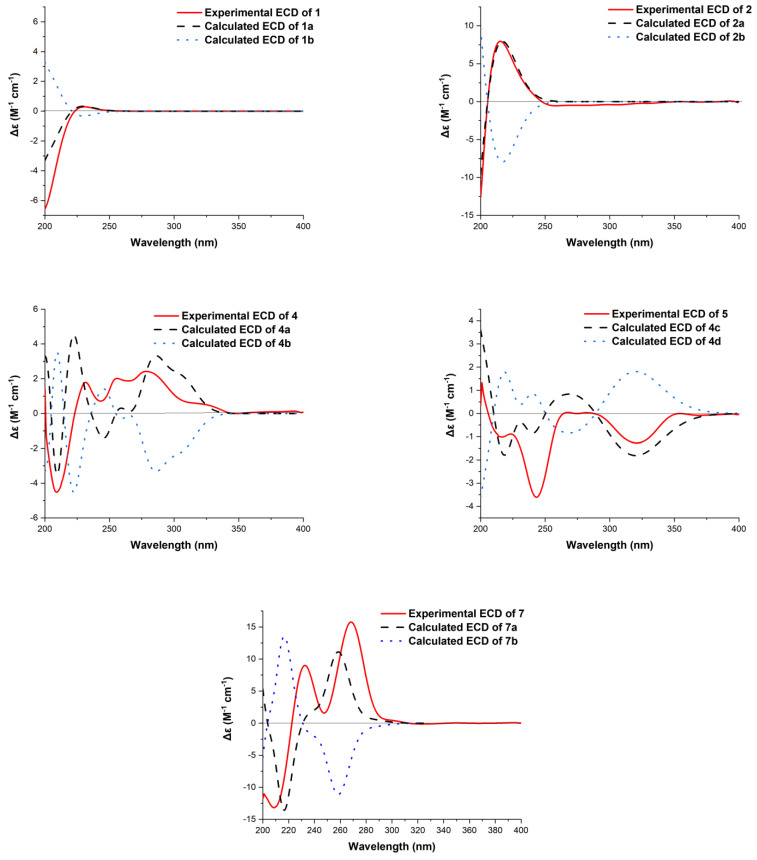
Calculated and experimental ECD spectra of **1**, **2, 4**, **5** and **7**.

The relative configuration of **1** was assigned by Rotating Frame Overhauser Effect Spectroscopy (ROESY) experiment (Figure 2) and, by comparison, with the known compound penicichrysogene B (**3**). The large J value observed for H-3a/H-2 (12.0 Hz) indicated their *trans*-diaxial orientations. The ROESY correlations of H-5 with H-1a, H-3a, and H-9, and of H-3a with H_3_-18 suggested the *α*-orientated of these protons, whereas those of H-2 with H-1b and H_3_-20 indicated these protons were *β*-orientated. Additionally, the ROESY correlations of H-14 with H_2_-12 suggested the *E*-configuration of the Δ^13^ double bond. The absolute configuration of **1** was assessed by comparison of the experimental and simulated ECD spectra generated by the time-dependent density functional theory (TDDFT) for two enantiomers (2*R*,4*S*,5*R*,9*S*,10*R*)-**1** (**1a**) and (2*S*,4*R*,5*S*,9*R*,10*S*)-**1** (**1b**). The experimental ECD spectrum of **1** was nearly identical to the calculated ECD spectrum for **1a** (Figure 3), clearly indicating the 2*R*,4*S*,5*R*,9*S*,10*R* absolute configuration for **1**. Thus, the structure of **1** was elucidated, as depicted in Figure 1.

Hypoxyterpoid B (**2**) was obtained as a brown oil. Its molecular formula C_21_H_26_O_10_ was established by HRESIMS [M + H]^+^ at *m/z* 439.1605 (calcd for C_21_H_27_O_10_ 439.1604), indicating the nine degrees of unsaturation. Its ^1^H and ^13^C NMR data (Table 1) indicated the presence of five carbonyl carbons (*δ*_C_ 175.9, 173.5, 172.8, 172.6, and 167.7, respectively), two quaternary carbons, four olefinic carbons with two protonated, three sp^3^ methine carbons with one oxygenated, four sp^3^ methylene carbons, and three methyl carbons (one oxygenated). These data suggested that **2** was also a bicyclic compound. Detailed comparison of the ^1^H and ^13^C NMR data of **2** (Table 1, Figures S7 and S8) with those of **1** revealed the same fragment of C-11–C-16, which was confirmed by interpretation of the ^1^H-^1^H COSY and HMBC data (Figure 2, Figures S9 and S11). However, obvious differences in chemical shifts were also observed for some signals for rings A and B. Specifically, the resonances for methylene carbon C-6 (*δ*_H/C_ 1.70; 1.88/25.5) in ring B of **1** were replaced by the oxygenated methine carbon C-6 (*δ*_H/C_ 4.93/77.7), which was confirmed by the ^1^H-^1^H COSY correlations H-5/H-6/H-7 and the HMBC correlations from H-5 to C-9 and C-10, from H-6 to C-5 and C-10, from H_2_-17 to C-7, C-8 and C-9, and from H-9 to C-8 and C-10. Other HMBC correlations from H_2_-1 to C-2, C-5 and C-10, from H-5 to C-1, C-3, C-4 and C-10 and from H_3_-18 to C-3, C-4, C-5 and C-19, as well as the bicyclic feature of **2** established a seven-membered ring (ring A) with the methyl carbon C-18 and the carboxylic carbon C-19 attached at C-4. Additionally, HMBC correlations from H_2_-1 and H-9 to C-10 and C-20 suggested the carbonyl carbon C-20 was attached to C-10 directly. While HMBC correlation from H_3_-21 to C-20 located the methoxyl group at C-10. On the basis of these data, the gross structure of **2** was established as shown.

The ROESY correlations of H-7b with H-5, H-6 and H-9, and of H-6 with H_3_-18 revealed the cofacial relationships of these groups (Figure 2). The relative configuration for C-10 was proposed to be the same as **1** and penicichrysogene B (**3**) from the biosynthetic considerations. The absolute configuration for **2** was also proposed, by a comparison of the experimental and calculated ECD spectra, for the enantiomers (4*S*,5*R*,6*R*,9*S*,10*R*)-**2** (**2a**) and (4*R*,5*S*,6*S*,9*R*,10*S*)-**2** (**2b**). The result showed that the calculated spectrum of (4*S*,5*R*,6*R*,9*S*,10*R*)-**2a** agreed with the experimental one (Figure 3), indicating the absolute configuration of **2** to be 4*S*,5*R*,6*R*,9*S*,10*R.* Compound **2** is a rare type of new diterpenoid derivative with an anhydride moiety. There are few precedents, in the natural products literature, for this type of structures [26,27,28].

Hypoxymarin A (**4**) was obtained as a yellow oil. Its molecular formula C_14_H_16_O_5_ was established by HRESIMS analysis (*m/z* 263.0925 [M − H]^−^), indicating seven degrees of unsaturation. The IR spectrum of **4** at 3306, 1697, 1658, 1598 cm^−1^ showed the presence of hydroxy, ester carbonyl and aromatic groups. Analysis of its NMR data (Table 2, Figures S13 and S14) and revealed the presence of one exchangeable proton (*δ*_H_ 9.63), three methyl groups (including one methoxy), two sp^3^ methines (one oxygenated), eight aromatic or olefinic carbons (three protonated), and one carbonyl carbon. These data accounted for all ^1^H and ^13^C NMR resonances except for one unobserved exchangeable proton. The remaining two degrees of unsaturation suggested that **4** was a bicyclic compound. The ^1^H-^1^H correlations (Figure 3) of H-9 with H-10 and H_3_-11, and of H-10 with H_3_-12 allowed the assignment of the C-11–C-9–C-10–C-12 butan-2-ol subunit. The HMBC correlations (Figure 3) from H-4 (*δ*_H_ 6.22) to C-3 and C-9, H-9 to C-3 and C-4, and from H-10 and H_3_-11 to C-3 indicated that C-9 was directly attached to C-3 of the trisubstituted olefin C-3/C-4. Additional HMBC correlations from H-4 to C-1, C-4a, C-8a and C-5, H-5 to C-4, C-6, C-7, and C-8a, and from H-7 to C-1, C-5, C-6 (*δ*_C_ 164.7), C-8 and C-8a, as well as from the methoxy group H_3_-13 to C-8 established the isochromenone core structure, with the methoxy group carbon C-13 located at C-8. The remaining two exchangeable protons were located at C-6 and C-10 by default. Thus, the planar structure of **4** was established as shown (Figure 1).

The absolute configuration for **4** was also assigned by ECD calculations. The calculated ECD spectra were obtained by the time-dependent density functional theory (TD-DFT) at the B3LYP/6-311G (2d, p) level for four stereoisomers (9*S*,10*S*)-**4** (**4a**), (9*R*,10*R*)-**4** (**4b**), (9*R*,10*S*)-**4** (**4c**) and (9*S*,10*R*)-**4** (**4d**). The overall calculated ECD spectrum of **4a**–**4d** were generated according to Boltzmann weighting of the conformers. The experimental ECD spectrum of **4** was nearly identical to the calculated ECD spectrum for (9*S*,10*S*)-**4** (**4a**) (Figure 3), indicating the absolute configuration to be 9*S*,10*S*-**4**.

Hypoxymarin B (**5**) was obtained as a yellow oil. The molecular formula was determined as C_14_H_16_O_5_ (seven degrees of unsaturation) by HRESIMS (*m/z* 263.0924 [M − H]^−^), which is the same with that of **4**. The ^1^H and ^13^C NMR data (Table 2, Figures S19 and S20) and 2D NMR were very similar, indicating that **5** was a stereoisomer of **4**. Subsequent comparison of the experimental ECD spectra of **5**, and the calculated ECD spectrum for (9*R*,10*S*)-**4** (**4c**), unambiguously assigned its absolute configuration as 9*R*,10*S.* Thus, the structure of **5** was elucidated, as depicted in Figure 1.

The molecular formula of hypoxymarin C (**6**) was determined to be C_14_H_18_O_5_ (six degrees of unsaturation) by HRESIMS (*m/z* 267.1226 [M + H]^+^). In the ^1^H NMR spectrum (Table 3), the proton signals and the coupling constants at *δ*_H_ 7.12 (d, *J* = 8.8 Hz), 6.71 (d, *J* = 8.8 Hz) indicated the presence of a 1,2,3,6-tetrasubstituted benzene system. Analysis of its NMR data (Table 3, Figures S25 and S26) the presence of one methyl group, four methylenes, two oxygenated methines, six aromatic carbons (two protonated) and one carbonyl carbon (*δ*_C_ 170.8). The NMR data of **6** resembled those of aspergillumarin B (**10**) [21] except that the proton H-5 in **10** were replaced by hydroxyl group in **6**, which was confirmed by the chemical shift of C-5 (*δ*_C_ 146.3) and the HMBC correlations from H_2_-4 and H-6 to C-5. On the basis of these results, the planar structure of **6** was elucidated (Figure 1). In order to determine the absolute configuration of C-4′ at the side chain, a [Rh_2_(OCOCF_3_)_4_]-induced ECD experiment was applied (Figure S34). In the induced ECD spectrum, the positive Cotton effect, at 350 nm, indicated a 4′*S* configuration according to the bulkiness rule [29]. By comparison with the dihydroisocoumarin data described in the literature [20,21], the negative circular dichroism at 259 nm indicated the *R* configuration at C-3. Thus, the absolute configuration of **6** was established as 3*R*,4′*S*.

**Figure 4:**
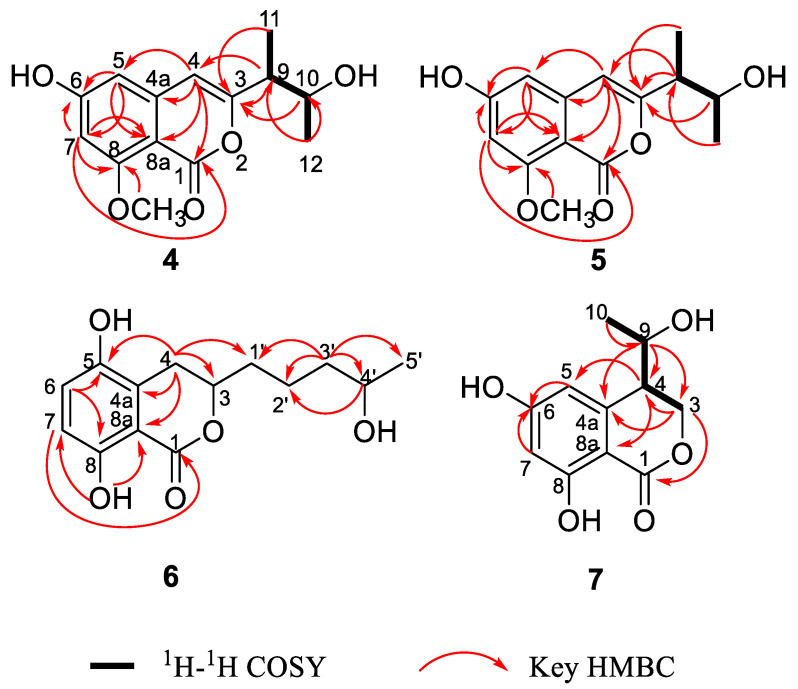
Key COSY and HMBC correlations of **4**–**7**.

Hypoxymarin D (**7**) was isolated as a yellow oil with the molecular formula assigned as C_11_H_12_O_5_ on the basis of its HREIMS (*m/z* 223.0614 [M − H]^−^), indicating six degrees of unsaturation. Analysis of the ^1^H and ^13^C NMR data (Table 3, Figures S28 and S29) revealed the presence of one methyl, one oxygenated methylene, two methines (one oxygenated), six aromatic carbons with two protonated, and one carbonyl carbon (*δ*_C_ 169.1). The ^1^H and ^13^C NMR data of **7** were almost consistent with penicimarin A (**8**). Further analysis of its ^1^H-^1^H COSY correlations (Figure 4) of H-9 with H_3_-10 and H-4, and of H_2_-3 with H-4, as well as the HMBC correlations (Figure 4) from H_3_-10 to C-9, from H-9 to C-3, C-4 and C-4a, from H_2_-3 to C-1, C-4 and C-4a, from H-4 to C-5 and C-8a, and from H-5 and H-6 to C-7 indicated that **7** and **8** share the same planar structure.

By treating **7** with Rh_2_(OCOCF_3_)_4_ in anhydrous CH_2_Cl_2_, a negative Cotton effect at 350 nm in the ECD spectrum was observed for the complex (Figure S34). Therefore, successful implementation of the bulkiness rule allowed C-9 to be assigned as *R*. The absolute configuration of C-4 was further determined by a comparison of the experimental and calculated ECD spectra. The calculated spectrum of (4*S*,9*R*)-**7a** agreed with the experimental one (Figure 3), indicating the absolute configuration of **7** to be 4*S*,9*R.*

The known compounds, penicichrysogene B (**3**) [19] penicimarin A (**8**) [20], aspergillumarin A (**9**) [21], aspergillumarin B (**10**) [21], 5-hydroxysescandelin (**11**) [22], sescandelin A (**12**) [23], and sescandelin B (**13**) [24] (Figure 1) were determined by comparison of their spectroscopic data with those in the literature.

The antibacterial activities of all isolated compounds were evaluated with *Bacillus subtilis*, *Escherichia coli*, and *Staphylococcus aureus*. However, these compounds showed no antibacterial activities against *B. subtilis*, *E. coli*, and *S. aureus* at the concentration of 200 μg/mL. All of the isolated compounds were also evaluated for radical-scavenging activity against 2,2-diphenyl-1-picrylhydrazyl (DPPH) radicals. Compounds **6** and **11** showed free radical scavenging activity with IC_50_ values of 15.36 ± 0.24 and 3.69 ± 0.07 μM, respectively (ascorbic acid as the positive control with IC_50_ values of 20.49 ± 0.43 μM). The α-glucosidase inhibitory effects of these compounds were evaluated along with the clinical *α*-glucosidase inhibitor acarbose. Compound **1** exhibited moderate inhibitory effects against *α*-glucosidase with IC_50_ value of 741.50 ± 2.83 μM (acarbose as positive control with IC_50_ value of 636.80 ± 1.49 μM).

Penicichrysogene B (**3**) was investigated in the anti-platelets assay and showed no inhibitory activities against AChE and BuChE [19]. Isocoumarin derivatives **8**–**13** was evaluated for antibacterial activities and cytotoxic activities in *vitro*, while only compounds **9** and **10** showed weak antibacterial activity against *Staphylococcus aureus* and *Bacillus subtilis* [20,21,22,23,24,30]. It is the first time, for **11**, that the antioxidant activity has been evaluated in our study.

## 3. Experimental Section

### 3.1. General Experimental Procedure

Optical rotations were measured with an Anton Paar MCP 200 Automatic Polarimeter (Anton Paar GmbH, Graz, Austria). Infrared spectra were obtained on a Nicolet IS5 FT-IR spectrophotometer (Thermo Scientific, Madison, WI, USA). The NMR data were measured on a Bruker Avance-500 MHz spectrometer (Bruker, Rheinstetten, Germany). The UV data were recorded on a Thermo Scientific Genesys 10S spectrophotometer (Thermo Scientific, Madison, WI, USA). ECD spectra were acquired on an Applied Photophysics Chirascan spectropolarimeter (Applied Photophysics Ltd., Leatherhead, UK). Mass data were performed on an Agilent Accurate-Mass-Q-TOF LC/MS 6520 instrument (Agilent Technologies, Santa Clara, CA, USA). Preparative HPLC was conducted with an Agilent 1200 HPLC system using a C_18_ column (Reprosil-Pur Basic C_18_ column; 5 μm; 10 × 250 mm) with a flow rate of 2.0 mL/min. Preparative HPLC was performed on the Waters system, using a C_18_ column (SunFire C_18_ column; 5 μm; 19 × 250 mm), with a flow rate of 15 mL/min. The absorbance of contents, in the 96-well clear plate, was detected by a SpectraMax Paradigm microplate reader (Molecular Devices, Sunnyvale, CA, USA).

### 3.2. Strain and Fermentation

*Hypoxylon* sp. (Hsl2-6) was isolated from the branches of *Bruguiera gymnorrhiza* collected in Beilun River Mouth, Fangchenggang City, Guangxi Province, P. R. China. The isolated strain was identified by sequence analysis (Genbank Accession Number MN547522) of the rDNA internal transcribed spacer (ITS) region. The strain was first placed on potato-dextrose agar medium and cultured at 25 °C for 7 days. Then the agar was cut into grain size to inoculate in four conical flasks (500 mL), each containing 100 mL autoclave sterilized potato dextrose broth. Seed culture medium was cultured at 25 °C and 180 rpm for 7 days. Finally, Erlenmeyer flasks (500 mL) containing 80 g of rice, 120 mL of distilled H_2_O, and 10.0 mL seed culture incubated at 25 °C for 30 days.

### 3.3. Extraction and Isolation

At the end of the fermentation, the rice culture was extracted with EtOAc (three times, each 12 L) and vacuum-dried to afford the crude extract (30.0 g). The extract was fractionated by ODS column chromatography (CC), eluted with 10–100% MeOH to afford twelve fractions (Frs.1–12). The fractions Frs.1–5 (8.7 g) were combined and subjected to silica gel CC and eluted with CHCl_3_/MeOH (20:1–5:1) to afford five subfractions (Frs.1.1–1.5). The subfraction Fr.1.1 (1.8 g) was subjected to Sephadex LH-20 CC, eluted with CHCl_3_/MeOH (2:1) to generate five fractions (Frs.1.1.1–1.1.5). Fr.1.1.2 (0.7 g) was loaded on normal pressure silica gel CC with petroleum ether (PE)/acetone (200:1–5:1) to yield Frs.1.1.2.1–1.1.2.6. The Fr.1.1.2.5 was further purified by RP HPLC (50% MeOH in H_2_O with 0.1% HCOOH; 15.0 mL/min) to afford **9** (9.0 mg, *t*_R_ 29.3 min). The subfraction Fr.1.2 (2.0 g) was loaded on Sephadex LH-20 CC, eluted with CHCl_3_/MeOH (2:1), to afford five tertiary fractions (Frs.1.2.1–1.2.5). The precipitation **11** (30.0 mg) was obtained from Fr.1.2.2. The remaining of Fr.1.2.2. was then purified by RP HPLC (40% MeOH in H_2_O with 0.1% HCOOH; 15.0 mL/min) to afford **13** (2.0 mg, *t*_R_ 19.0 min) and **12** (3.0 mg, *t*_R_ 20.8 min). Fr.1.2.4 (0.8 g) was fractionated by normal pressure silica gel CC with PE/acetone (15:1–5:1) to afford three fractions (Frs.1.2.4.1–1.2.4.3). Fr.1.1.4.2 was purified by RP HPLC (36% MeOH in H_2_O with 0.1% HCOOH; 15.0 mL/min) to afford **7** (5.0 mg, *t*_R_ 18.0 min) and **8** (8.1 mg, *t*_R_ 21.0 min). The subfraction Fr.1.3 (100.2 mg) was loaded on Sephadex LH-20 CC eluting with CHCl_3_/MeOH (2:1) to afford seven fractions (Frs.1.3.1–1.3.7). Fr.1.3.7 was further purified by RP HPLC (42% MeOH in H_2_O with 0.1% HCOOH; 2.0 mL/min) to afford **6** (5.7 mg, *t*_R_ 24.0 min). Fr.1.5 (1.6 g) was loaded on Sephadex LH-20 CC eluted with CHCl_3_/MeOH (2:1) to afford Frs.1.5.1–1.5.3. Fr.1.5.2 was further purified by RP HPLC (45% MeOH in H_2_O with 0.1% HCOOH; 15.0 mL/min) to afford **4** (5.0 mg, *t*_R_ 15.0 min) and **5** (2.5 mg, *t*_R_ 18.0 min). The subfraction Fr.6.2 was purified by RP HPLC (50% MeOH in H_2_O with 0.1% HCOOH; 2.0 mL/min) to afford **2** (9.2 mg, *t*_R_ 23.0 min) and **10** (4.3 mg, *t*_R_ 31.5 min). The fraction Fr.8 was purified by RP HPLC (60% MeOH in H_2_O with 0.1% HCOOH; 2.0 mL/min) to afford **1** (3.0 mg, *t*_R_ 31.9 min) and **3** (3.0 mg, *t*_R_ 45.5 min).

Hypoxyterpoid A (**1**): brown oil; [α${]}_{D}^{25}$ +28.8 (*c* 0.15, MeOH); UV (MeOH) *λ*_max_ (log *ε*): 200 (2.44) nm; IR (neat) *ν*_max_ 2941, 2851, 1703, 1644, 1446, 1384, 1231, 1155, 1025, 1003, 889, 824, 762 cm^−1^; CD (MeOH) *λ*_max_ (Δ*ε*) 202 (−0.56), 222 (+1.96), 287 (−0.33); ^1^H and ^13^C NMR data (500 MHz, DMSO-*d*_6_) see Table 1; HRESIMS: *m/z* 373.1995 [M + Na]^+^ (calcd for C_20_H_30_O_5_Na 373.1991).

Hypoxyterpoid B (**2**): brown oil; [α${]}_{D}^{25}$ +28.2 (*c* 0.85, MeOH); UV (MeOH) *λ*_max_ (log *ε*): 200 (3.02); 210 (2.20) nm; IR (neat) *ν*_max_ 2952, 1779, 1726, 1644, 1435, 1219, 1165, 1115, 1008, 968, 907 cm^−1^; CD (MeOH) *λ*_max_ (Δ*ε*) 200 (−12.61), 215 (+7.96); ^1^H and ^13^C NMR data (500 MHz, Acetone-*d*_6_) see Table 1; HRESIMS: *m/z* 439.1605 [M + H]^+^ (calcd for C_21_H_27_O_10_ 439.1604).

Hypoxymarin A (**4**): Yellow oil; [α${]}_{D}^{25}$ −0.6 (*c* 0.50, MeOH); UV (MeOH) *λ*_max_ (log *ε*): 245 (0.69); 324 (0.13) nm; IR (neat) *ν*_max_ 3306, 2975, 1697, 1658, 1598, 1440, 1369, 1237, 1200, 1174, 1123, 1074, 1026, 947, 907, 855 cm^−1^; CD (MeOH) *λ*_max_ (Δ*ε*) 200 (+1.47), 209 (−4.52), 231 (+1.79), 278 (+2.42) nm; ^1^H and ^13^C NMR data (500 MHz, Acetone-*d*_6_) see Table 2; HRESIMS *m/z* 263.0925 [M − H]^−^ (calcd for C_14_H_15_O_5_, 263.0919).

Hypoxymarin B (**5**): Yellow oil; [α${]}_{D}^{25}$ + 26.4 (*c* 0.25, MeOH); UV (MeOH) *λ*_max_ (log *ε*): 245 (1.27); 324 (0.21) nm; IR (neat) *ν*_max_ 3420, 2976, 1693, 1656, 1598, 1496, 1441, 1369, 1302, 1238, 1199, 1176, 1121, 1078, 1026, 966, 833, 793cm^−1^; CD (MeOH) *λ*_max_ (Δ*ε*) 201 (+1.33), 243 (−3.61), 320 (−1.27) nm; ^1^H and ^13^C NMR data (500 MHz, Acetone-*d*_6_) see Table 2; HRESIMS *m/z* 263.0924 [M − H]^–^ (calcd for C_14_H_1__5_O_5_, 263.0919).

Hypoxymarin C (**6**): White powder; [α${]}_{D}^{25}$ –37.2 (*c* 0.50, MeOH); UV (MeOH) *λ*_max_ (log *ε*): 228 (3.06); 247 (1.73) nm; IR (neat) *ν*_max_ 3218, 2962, 2929, 2860, 1657, 1590, 1471, 1406, 1354, 1286, 1127, 1065, 1013, 944, 820, 701 cm^−1^; CD (MeOH) *λ*_max_ (Δ*ε*) 200 (−2.01), 221 (+1.96), 260 (−3.18), 283 (+0.75) nm; ^1^H and ^13^C NMR data (500 MHz, Acetone-*d*_6_) see Table 3; HRESIMS *m/z* 267.1226 [M + H]^+^ (calcd for C_14_H_19_O_5_, 267.1227).

Hypoxymarin D (**7**): Yellow oil; [α${]}_{D}^{25}$ −30.8 (*c* 0.50, MeOH); UV (MeOH) *λ*_max_ (log *ε*): 200 (2.54), 268 (0.36), 302 (0.19) nm; IR (neat) *ν*_max_ 3140, 2977, 1662, 1628, 1466, 1400, 1323, 1248, 1167, 1130, 1092, 1047, 1024, 1001, 852, 824, 761 cm^−1^; CD (MeOH) *λ*_max_ (Δ*ε*) 209 (−13.17), 233 (+9.00), 248 (+1.59), 268 (+15.77) nm; ^1^H and ^13^C NMR data (500 MHz, Acetone-*d*_6_) see Table 3; HRESIMS *m/z* 223.0614 [M − H]^−^ (calcd for C_1__1_H_11_O_5_, 223.0606).

### 3.4. Antimicrobial Assay

All compounds were tested for antibacterial activities against three pathogenic bacteria (*S. aureus* CGMCC1.2465, *B. subtilis* ATCC6663, and *E. coli* CGMCC1.2340) in 96-well microplates, according to the method of Fan et al. [31]. Ampicillin was used as positive controls, and DMSO was used as a negative control. Repeated all experiments three times.

### 3.5. DPPH Assay

The scavenging activity of DPPH radical was measured as described in previous literature [31]. The scavenging ability was calculated as follows: scavenging ability (%) = (1-A517 of sample/A517 of control) × 100. Ascorbic acid was used as a positive control, and DMSO was used as a negative control. All experiments were repeated three times.

### 3.6. α-Glucosidase Inhibitory Assay

First, 10.88 U/mL of α-glucosidase from *Saccharomyces cerevisiae* was diluted with 0.1 M phosphate buffer to 0.2 U/mL. The assay was performed in a 50 µL reaction system, which contains 10 µL of diluted enzyme solution, 20 µL of 0.1 M phosphate buffer, and 10 µL of dimethyl sulfoxide (DMSO) or sample (dissolved in DMSO). After 10 min of incubation in 96-well plates at 37 °C, a 10 μL portion of 4 mM 4-nitrophenyl-α-d-glucopyranoside (PNPG) was added as a substrate to begin the enzymatic reaction (final concentrations of 50, 100, 200, 400, 800 μM). The plate was incubated for an additional 30 min at 37 °C, and the reaction was quenched by adding 50 μL of 0.2 M Na_2_CO_3_. The optical density (OD) was measured at an absorbance wavelength of 405 nm using a Spectra Max 190 microplate reader (Molecular Devices, Sunnyvale, CA, USA). Each assay was repeated three times, and acarbose was used as the positive control.

### 3.7. Rh_2_(OCOCF_3_)_4_-Induced ECD Experiments of ***6*** and ***7***

The sample of **6** and **7** (0.5 mg) was dissolved in a dry solution of the stock [Rh_2_(OCOCF_3_)_4_] complex (1.0 mg) in CDCl_3_ (200 μL) and was received, to CD measurements, at a concentration of 2.5 mg/mL. The first ECD spectrum was recorded immediately after mixing, and its time evolution was monitored until stationary (30 min after mixing). The inherent CD was subtracted. The absolute configurations of the secondary alcohols in **6** and **7** were determined [29].

### 3.8. ECD Calculation

Conformational analyses for compound **1**, **2**, **4**, and **7** were performed using Maestro 10.2 in the OPLS3 molecular mechanics force-field within an energy window of 3.0 kcal/mol. All of the conformers were then further optimized by the DFT methods at the B3LYP/6-311G(2d,p) level in the Gaussian 09 software package, respectively [32]. The TD-DFT methods at the B3LYP/6-311G(2d,p) were applied to calculate the 60 lowest electronic transitions, which obtained conformers in vacuum, respectively. The Gaussian function was applied to simulate the ECD spectrum of the conformers. The calculated ECD spectra were obtained according to the Boltzmann weighting of each conformer’s ECD spectrum in MeOH solution.

## 4. Conclusions

In conclusion, two new diterpenoids, hypoxyterpoids A and B (**1** and **2**), and four new isocoumarin derivatives, hypoxymarins A–D (**4**–**7**), together with seven known metabolites (**3** and **8**–**13**), were isolated from the fermentation broth of the mangrove-derived fungus *Hypoxylon* sp. The structures of the new compounds were elucidated on the basis of NMR spectroscopic and mass spectrometric analysis. The absolute configurations of compounds **1**, **2**, **4**, **5**, and **7** were determined by comparison of experimental and calculated ECD spectra, and the absolute configurations of C-4′ in compound **6** and C-9 in compound **7** were determined by [Rh_2_(OCOCF_3_)_4_]-induced ECD spectra. Compound **1** showed moderate *α*-glucosidase inhibitory activities with IC_50_ values of 741.5 ± 2.83 μM. Compounds **6** and **11** exhibited potent DPPH scavenging activities with IC_50_ values of 15.36 ± 0.24 and 3.69 ± 0.07 μM, respectively. Our findings also suggest that the fungal genus *Hypoxylon* spp. are a rich source of bioactive secondary metabolites, and thus worthy of in-depth investigations.

## Figures and Tables

**Table 1:** ^1^H NMR and ^13^C NMR data (500 and 125 MHz) for **1** (in DMSO-*d*_6_) and **2** (in Acetone-*d*_6_).

Position	1		2	
	*δ*_C_	*δ*_H_ (*J* in Hz)	*δ*_C_	*δ*_H_ (*J* in Hz)
1a	48.0, CH_2_	0.87, td (12, 3)	35.8, CH_2_	2.72, d (18.3)
1b		1.96, m		3.11, d (18.3)
2	62.9, CH	3.88, tt (4.2, 12)	172.8, C	
3a	46.9, CH_2_	0.87, td (12, 3)	173.5, C	
3b		2.19, m		
4	44.2, qC		55.0, C	
5	54.5, CH	1.26, m	54.7, CH	3.50, d (11.3)
6a	25.5, CH_2_	1.70, m	77.7, CH	4.93, m
6b		1.88, m		
7a	38.0, CH_2_	1.84, m	42.4, CH_2_	2.38, m
7b		2.34, m		3.05, dd (4.5, 11.3)
8	147.7, qC		142.7, C	
9	54.6, CH	1.60, m	47.4, CH	3.18, m
10	40.9, C		51.8, C	
11a	21.5, CH_2_	1.44, m	24.1, CH_2_	1.47, m
11b		1.62, m		1.77, m
12a	38.9, CH_2_	2.21, m	40.1, CH_2_	2.10, m
12b		1.94, m		2.34, m
13	158.6, C		160.1, C	
14	116.8, CH	5.54, s	116.5, CH	5.65, s
15	167.6, C		167.7, C	
16	18.3, CH_3_	2.07, s	18.7, CH_3_	2.13, s
17a	106.8, CH_2_	4.51, s	113.7, CH_2_	4.91, m
17b		4.88, s		5.21, s
18	28.7, CH_3_	1.15, s	13.3, CH_3_	1.25, s
19	178.3, C		175.9, C	
20	13.5, CH_3_	0.54, s	172.6, C	
21			52.0, CH_3_	3.67, s

**Table 2:** ^1^H NMR and ^13^C NMR data (500 and 125 MHz) for **4** and **5** in Acetone-*d*_6_.

Position	4		5	
	*δ*_C_	*δ*_H_ (*J* in Hz)	δ_C_	*δ*_H_ (*J* in Hz)
1	158.6, C		158.6, C	
3	161.6, C		161.3, C	
4	103.5, CH	6.22, s	103.8, CH	6.20, s
4a	143.2, C		143.3, C	
5	103.6, CH	6.43, s	103.5, CH	6.42, s
6	164.6, C		164.6, C	
7	99.4, CH	6.51, s	99.4, CH	6.51, s
8	164.7 C		164.7, C	
8a	102.8, C		102.9, C	
9	46.4, CH	2.45, m	46.7, CH	2.53, m
10	69.1, CH	3.98, m	69.3, CH	3.98, m
11	13.7, CH_3_	1.26, d (7.0)	14.2, CH_3_	1.19, d (7.0)
12	22.0, CH_3_	1.16, d (6.3)	20.7, CH_3_	1.17, d (6.2)
13	56.2, CH_3_	3.87, s	56.3, CH_3_	3.87, s
OH-6		9.63, br s		9.59, br s

**Table 3:** ^1^H NMR and ^13^C NMR data (500 and 125 MHz) for **6** (in Acetone-*d*_6_) and **7** (in DMSO-*d*_6_).

Position	6		Position	7	
	*δ*_C_	*δ*_H_ (*J* in Hz)		*δ*_C_	*δ*_H_ (*J* in Hz)
1	170.8, C		1	169.1, C	
3	80.5, CH	4.63, m	3	68.3, CH_2_	4.51, dd (4.1, 11.6) 4.61, dd (1.8, 11.6)
4	27.4, CH_2_	2.68, dd (3.4, 16.8) 3.19, dd (10.6, 16.8)	4	43.2, CH	2.79, m
4a	125.6, C		4a	143.0, C	
5	146.3, C		5	108.2, CH	6.26, d (2.2)
6	124.7, CH	7.12, d (8.8)	6	164.5, C	
7	116.1, CH	6.71, d (8.8)	7	101.2, CH	6.19, d (2.2)
8	156.2, C		8	163.1, C	
8a	109.3, C		8a	100.1, C	
1′	35.7, CH_2_	1.79, m 1.89, m	9	68.0, CH	3.85, m
2′	22.0, CH_2_	1.62, m	10	19.9, CH_3_	1.04, d (6.2)
3′	39.3, CH_2_	1.48, m	8-OH		11.16, s
4′	67.4, CH	3.77, m			
5′	24.1, CH_3_	1.14, d (6.2)			
8-OH		10.6, s			

## Data Availability

Data is contained within the article or supplementary material.

## Supplementary Materials

The following are available online at https://www.mdpi.com/article/10.3390/md19070362/s1, Figures S1–S33: 1D and 2D NMR for compounds **1****, 2** and **4**–**7**; Figure S34: ECD spectra of the [Rh_2_(OCOCF_3_)_4_] complexes of compounds **6** and **7**; Figure S35: ECD conformers of **1**, **2**, **4** and **7**.
