# Supplementary material for: New Diterpenoids and Isocoumarin Derivatives from the Mangrove-Derived Fungus Hypoxylon sp
Source: Mar Drugs. 2021 Jun 24;19(7):362. doi: 10.3390/md19070362 (PMC8305793; doi:10.3390/md19070362)
Supplement: Supplementary file 1 [file marinedrugs-19-00362-s001.zip › marinedrugs-1269425-supplementary.pdf]

# New Diterpenoids and Isocoumarin Derivatives from the Mangrove-Derived Fungus *Hypoxylon* sp.

Bolin Hou [1,†], Sushi Liu [1,2,†], Ruiyun Huo [1], Yueqian Li [1,3], Jinwei Ren [1], Wenzhao Wang [1], Tao Wei [3], Xuejun Jiang [1], Wenbing Yin [1], Hongwei Liu [1], Ling Liu [1,*], Erwei Li [1,3,*]

[1] State Key Laboratory of Mycology, Institute of Microbiology, Chinese Academy of Sciences, Beijing 100101, China; houbl@im.ac.cn (B.H.); liuss@im.ac.cn (S.L.); huory@im.ac.cn (R.H.); yqli19@lzu. edu. cn (Y.L.); renjw@im.ac.cn (J.R.); wangwz@im.ac.cn (W.W.); jiangxj@im.ac.cn (X.J.); yinwb@im.ac.cn (W.Y.); liuhw@im.ac.cn (H.L.)

[2] Beijing Key Laboratory of Bioactive Substance and Functional Foods, Beijing Union University, Beijing 100191, China; weitao@buu.edu.cn (W.T.)

[3] Institutional Center for Shared Technologies and Facilities, Institute of Microbiology, Chinese Academy of Sciences, Beijing 100101, China

* Correspondence: liul@im.ac.cn (L.L); liew@im.ac.cn (E.L.); Tel.: +86-10-6480-7043 (L.L.); +86-10-6480-6141 (E.L.)

† These authors contributed equally to this paper.

# Table of contents


| List | Content | Page |
|---|---|---|
| **Figure S1.** | $^{1}$H NMR spectrum of hypoxyterpoid A (**1**; 500 MHz, DMSO-$d_6$) | 4 |
| **Figure S2.** | $^{13}$C NMR spectrum of hypoxyterpoid A (**1**; 125 MHz, DMSO-$d_6$) | 4 |
| **Figure S3.** | $^{1}$H-$^{1}$H COSY spectrum of hypoxyterpoid A (**1**; 500 MHz, DMSO-$d_6$) | 5 |
| **Figure S4.** | HSQC spectrum of hypoxyterpoid A (**1**; 500 MHz, DMSO-$d_6$) | 5 |
| **Figure S5.** | HMBC spectrum of hypoxyterpoid A (**1**; 500 MHz, DMSO-$d_6$) | 6 |
| **Figure S6.** | ROESY spectrum of hypoxyterpoid A (**1**; 500 MHz, DMSO-$d_6$) | 6 |
| **Figure S7.** | $^{1}$H NMR spectrum of hypoxyterpoid B (**2**; 500 MHz, Acetone-$d_6$) | 7 |
| **Figure S8.** | $^{13}$C NMR spectrum of hypoxyterpoid B (**2**; 125 MHz, Acetone-$d_6$) | 7 |
| **Figure S9.** | $^{1}$H-$^{1}$H COSY spectrum of hypoxyterpoid (**2**; 500 MHz, Acetone-$d_6$) | 8 |
| **Figure S10.** | HSQC spectrum of hypoxyterpoid B (**2**; 500 MHz, Acetone-$d_6$) | 8 |
| **Figure S11.** | HMBC spectrum of hypoxyterpoid B (**2**; 500 MHz, Acetone-$d_6$) | 9 |
| **Figure S12.** | ROESY spectrum of hypoxyterpoid B (**2**; 500 MHz, Acetone-$d_6$) | 9 |
| **Figure S13.** | $^{1}$H NMR spectrum of hypoxymarin A (**4**; 500 MHz, Acetone-$d_6$) | 10 |
| **Figure S14** | $^{13}$C NMR spectrum of hypoxymarin A (**4**; 125 MHz, Acetone-$d_6$) | 10 |
| **Figure S15.** | $^{1}$H-$^{1}$H COSY spectrum of hypoxymarin A (**4**; 500 MHz, Acetone-$d_6$) | 11 |
| **Figure S16.** | HSQC spectrum of hypoxymarin A (**4**; 500 MHz, Acetone-$d_6$) | 11 |
| **Figure S17.** | HMBC spectrum of hypoxymarin A (**4**; 500 MHz, Acetone-$d_6$) | 12 |
| **Figure S18.** | ROESY spectrum of hypoxymarin A (**4**; 500 MHz, Acetone-$d_6$) | 12 |
| **Figure S19.** | $^{1}$H NMR spectrum of hypoxymarin B (**5**; 500 MHz, Acetone-$d_6$) | 13 |
| **Figure S20.** | $^{13}$C NMR spectrum of hypoxymarin B (**5**; 125 MHz, Acetone-$d_6$) | 13 |
| **Figure S21.** | $^{1}$H-$^{1}$H COSY spectrum of hypoxymarin B (**5**; 500 MHz, Acetone-$d_6$) | 14 |
| **Figure S22.** | HSQC spectrum of hypoxymarin B (**5**; 500 MHz, Acetone-$d_6$) | 14 |
| **Figure S23.** | HMBC spectrum of hypoxymarin B (**5**; 500 MHz, Acetone-$d_6$) | 15 |
| **Figure S24.** | ROESY spectrum of hypoxymarin B (**5**; 500 MHz, Acetone-$d_6$) | 15 |
| **Figure S25.** | $^{1}$H NMR spectrum of hypoxymarin C (**6**; 500 MHz, Acetone-$d_6$) | 16 |

| | | |
|---|---|---|
| **Figure S26.** | $^{13}C$ NMR spectrum of hypoxymarin C (**6**; 125 MHz, Acetone-$d_6$) | 16 |
| **Figure S27.** | HMBC spectrum of hypoxymarin C (**6**; 500 MHz, Acetone-$d_6$) | 17 |
| **Figure S28.** | $^{1}H$ NMR spectrum of hypoxymarin D (**7**; 500 MHz, DMSO-$d_6$) | 17 |
| **Figure S29.** | $^{13}C$ NMR spectrum of hypoxymarin D (**7**; 125 MHz, DMSO-$d_6$) | 18 |
| **Figure S30.** | $^{1}H$-$^{1}H$ COSY spectrum of hypoxymarin D (**7**; 500 MHz, DMSO-$d_6$) | 18 |
| **Figure S31.** | HSQC spectrum of hypoxymarin D (**7**; 500 MHz, DMSO-$d_6$) | 19 |
| **Figure S32.** | HMBC spectrum of hypoxymarin D (**7**; 500 MHz, DMSO-$d_6$) | 19 |
| **Figure S33.** | ROESY spectrum of hypoxymarin D (**7**; 500 MHz, DMSO-$d_6$) | 20 |
| **Figure S34.** | ECD spectra of the [$Rh_2(OCOCF_3)_4$] complexes of compounds **6** and **7** with the intrinsic ECD spectrum subtracted | 21 |
| **Figure S35.** | ECD conformers of **1**, **2**, **4** and **7** | 22 |

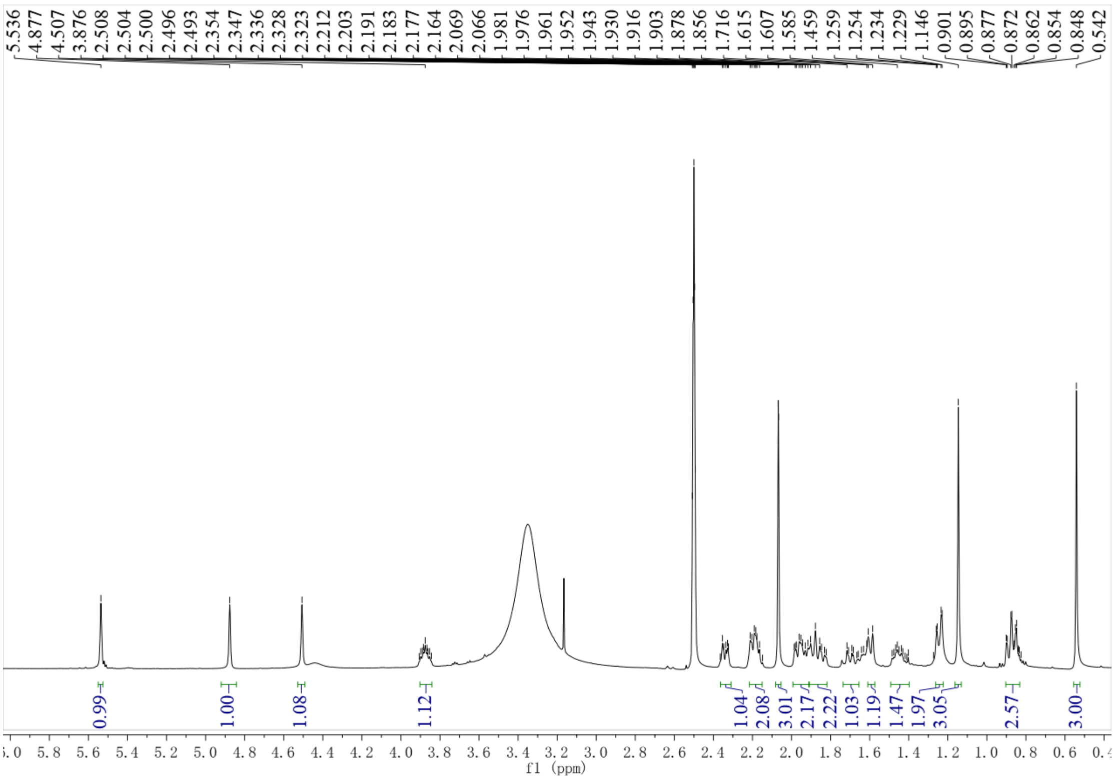

**Figure S1.** $^{1}$H NMR spectrum of hypoxyterpoid A (**1**; 500 MHz, DMSO-$d_6$)

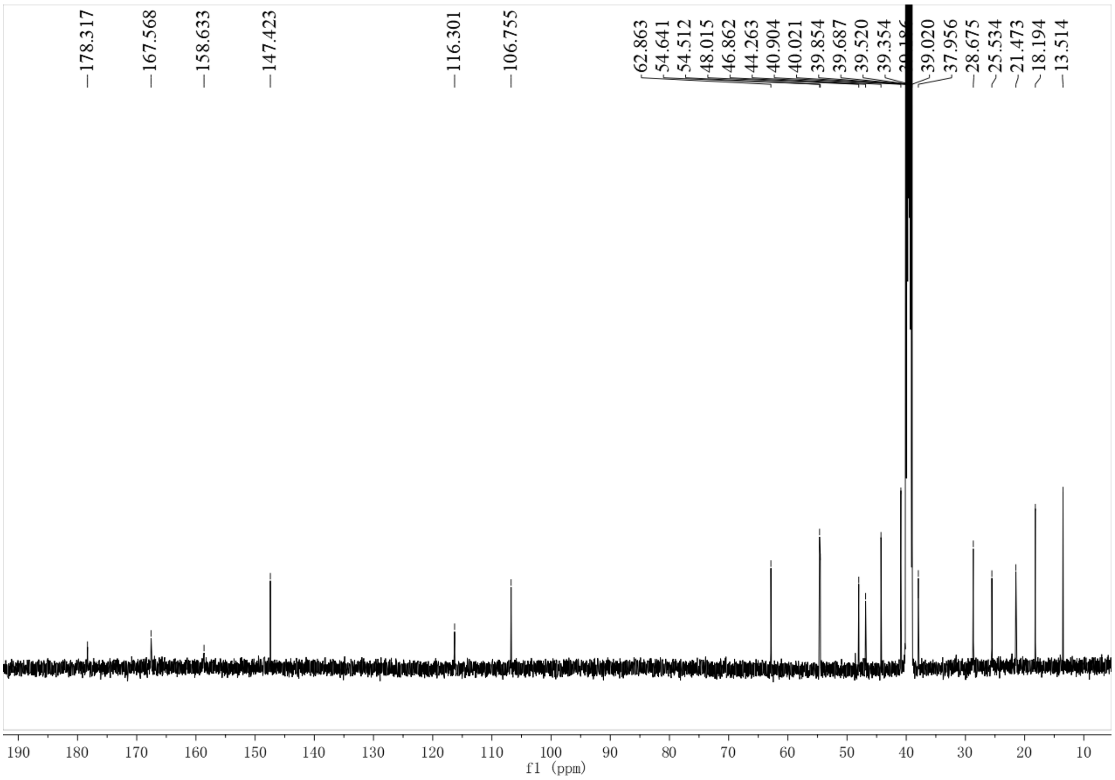

**Figure S2.** $^{13}$C NMR spectrum of hypoxyterpoid A (**1**; 125 MHz, DMSO-$d_6$)

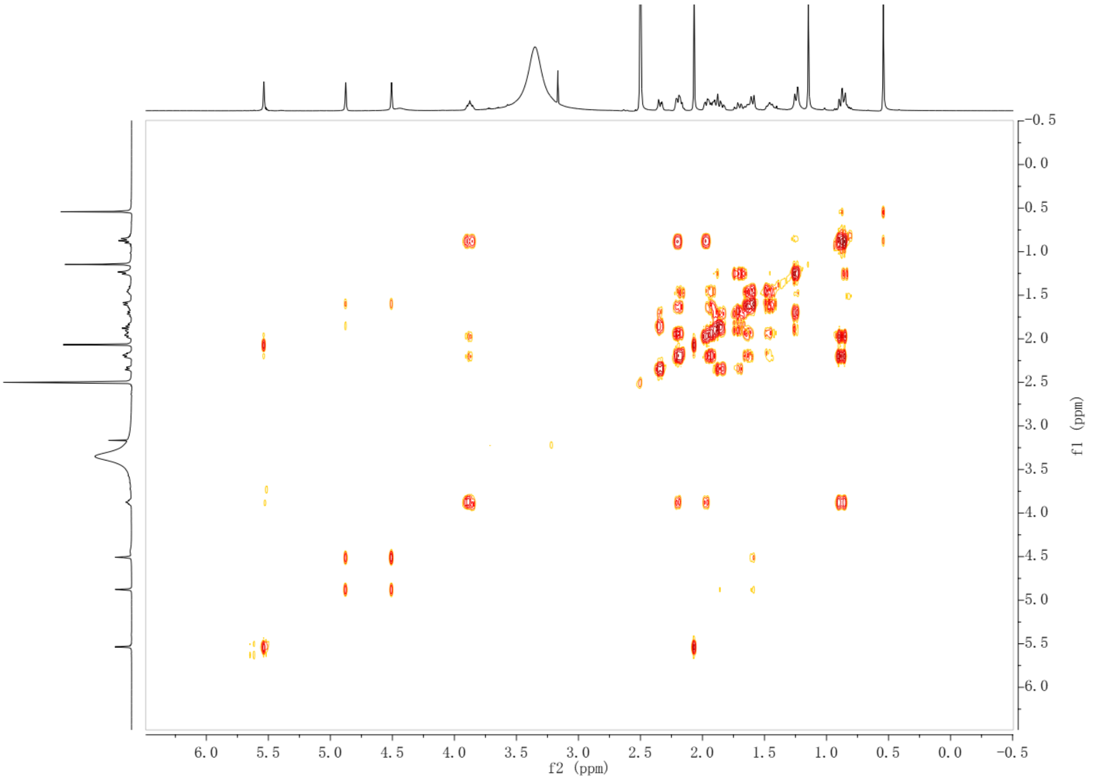


**Figure S3.** $^{1}H$-$^{1}H$ COSY spectrum of hypoxyterpoid A (**1**; 500 MHz, DMSO-$d_6$)

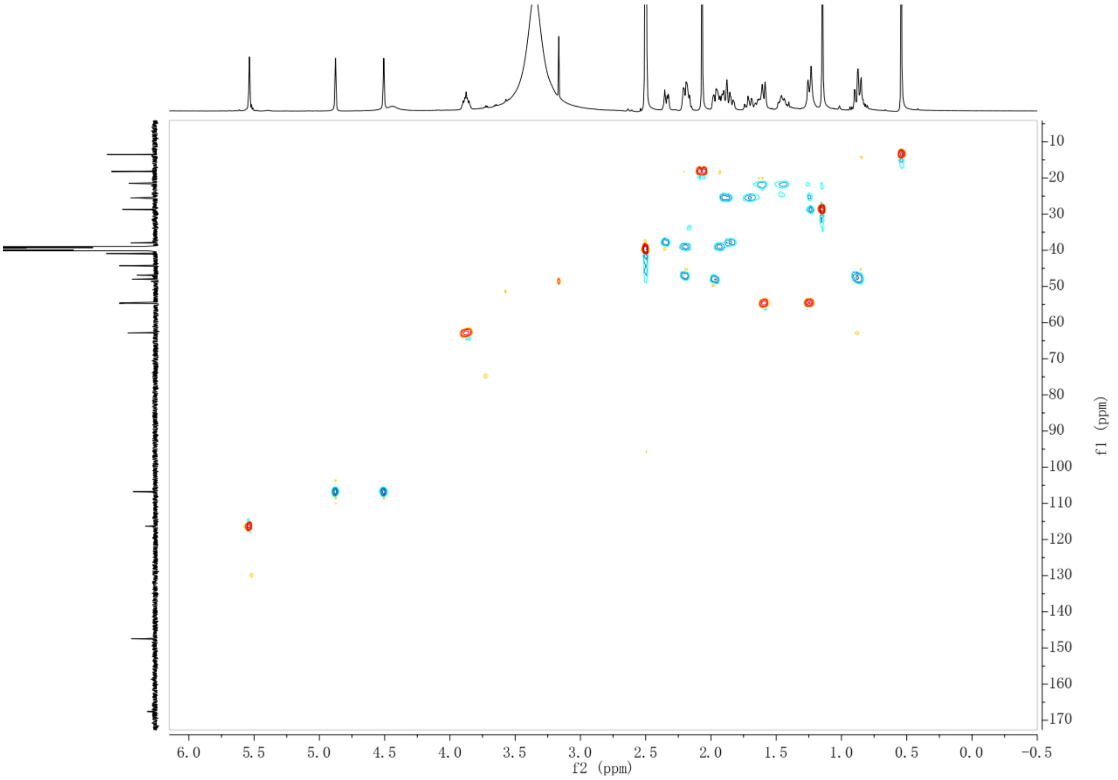


**Figure S4.** HSQC spectrum of hypoxyterpoid A (**1**; 500 MHz, DMSO-$d_6$)

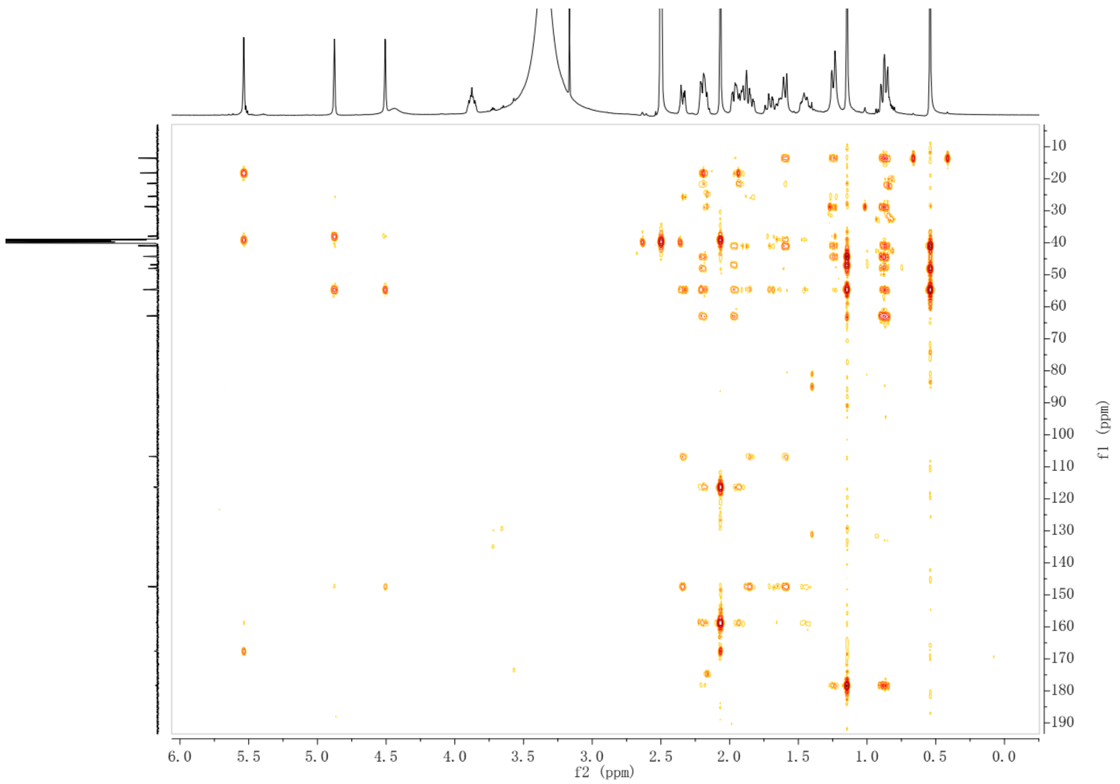


**Figure S5.** HMBC spectrum of hypoxyterpoid A (**1**; 500 MHz, DMSO-$d_6$)

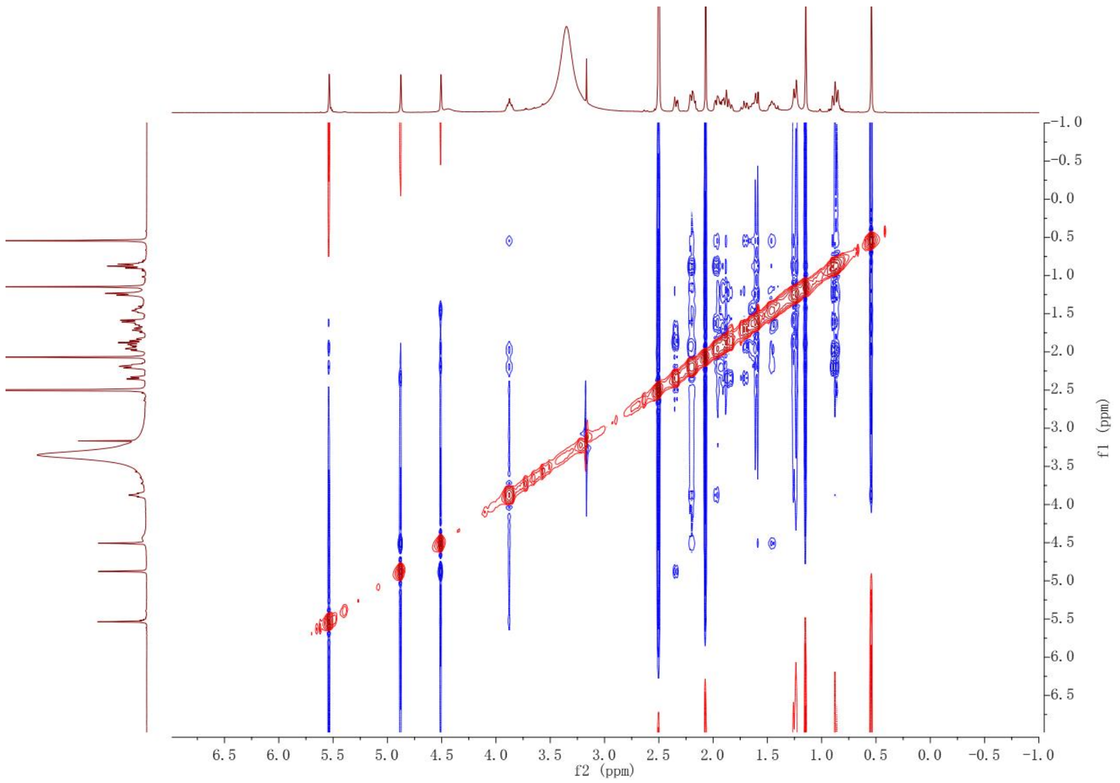


**Figure S6.** ROESY spectrum of hypoxyterpoid A (**1**; 500 MHz, DMSO-$d_6$)

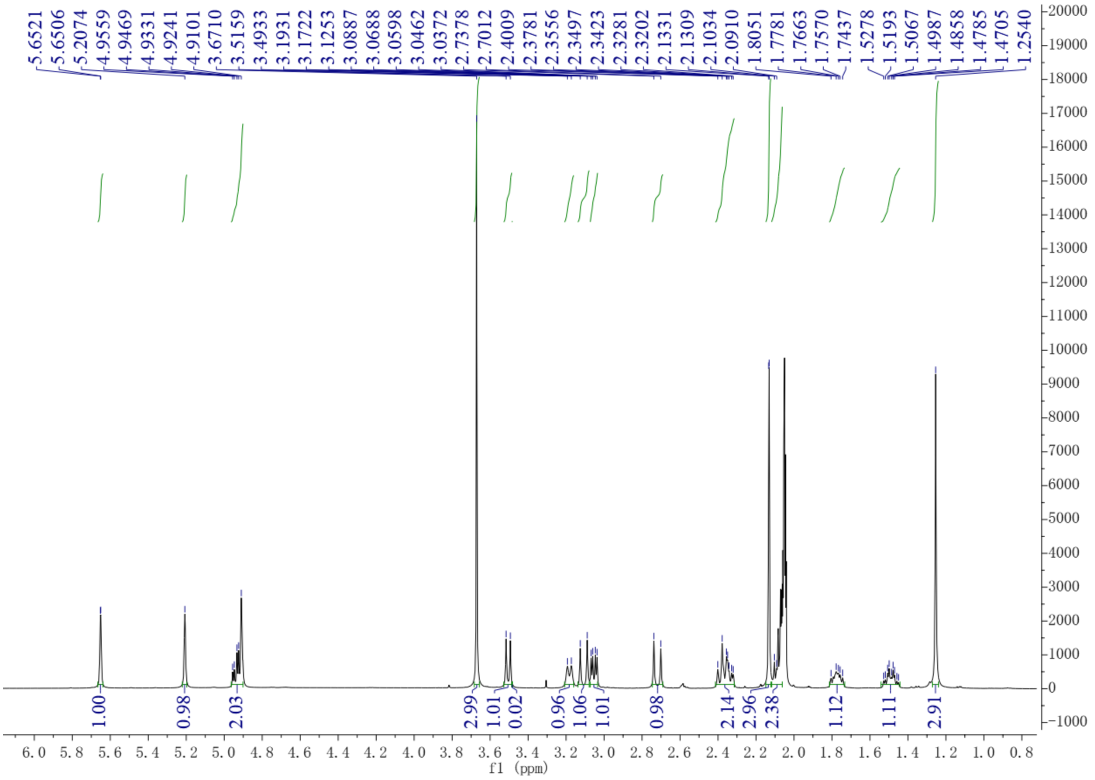


**Figure S7.** $^{1}$H NMR spectrum of hypoxyterpoid B (**2**; 500 MHz, Acetone-$d_6$)

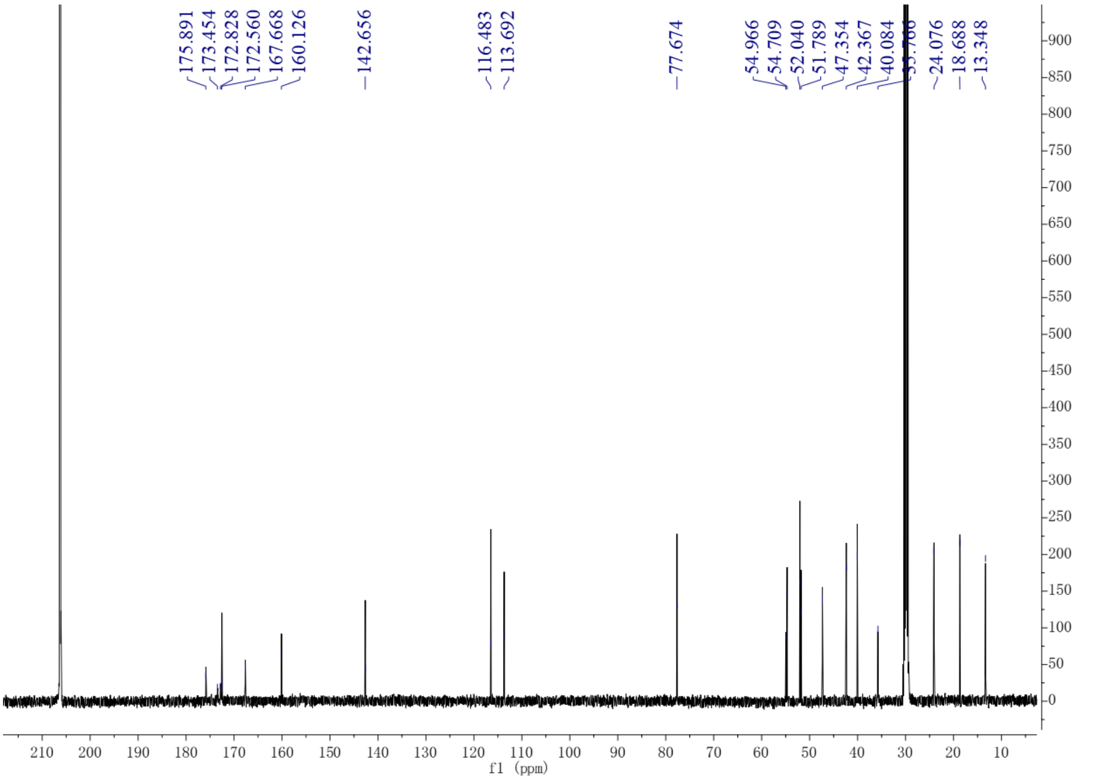


**Figure S8.** $^{13}$C NMR spectrum of hypoxyterpoid B (**2**; 500 MHz, Acetone-$d_6$)

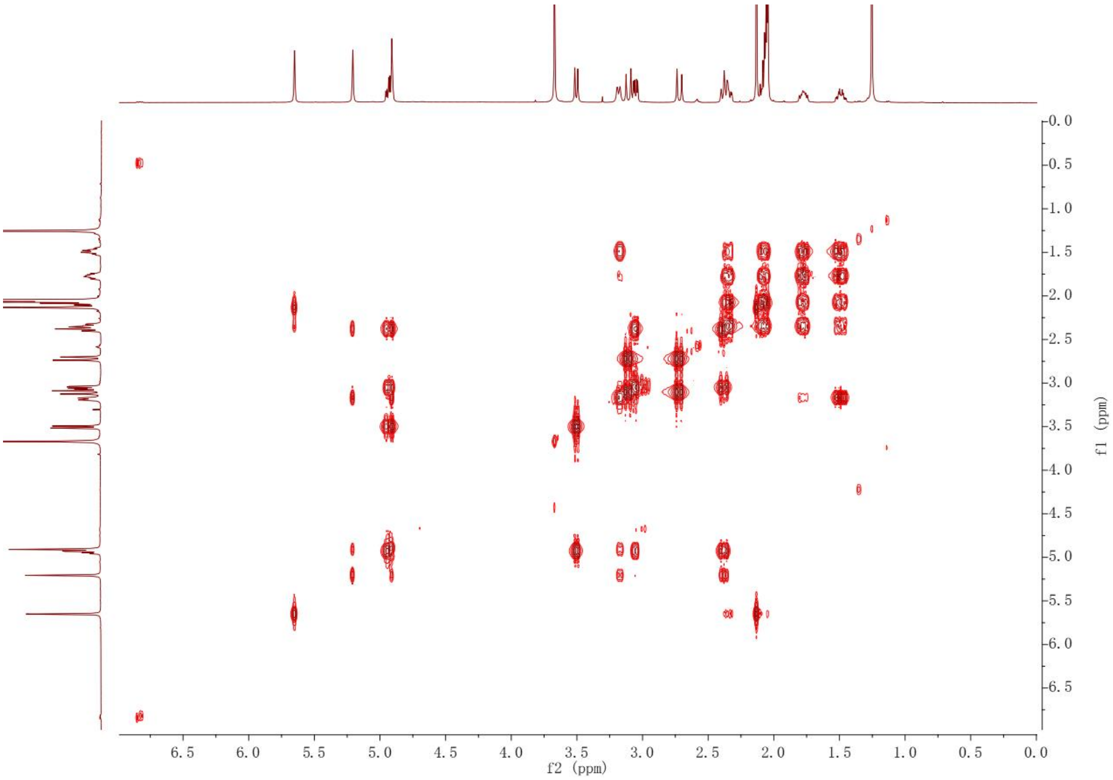


**Figure S9.** $^{1}H$-$^{1}H$ COSY spectrum of hypoxyterpoid B (**2**; 500 MHz, Acetone-$d_6$)

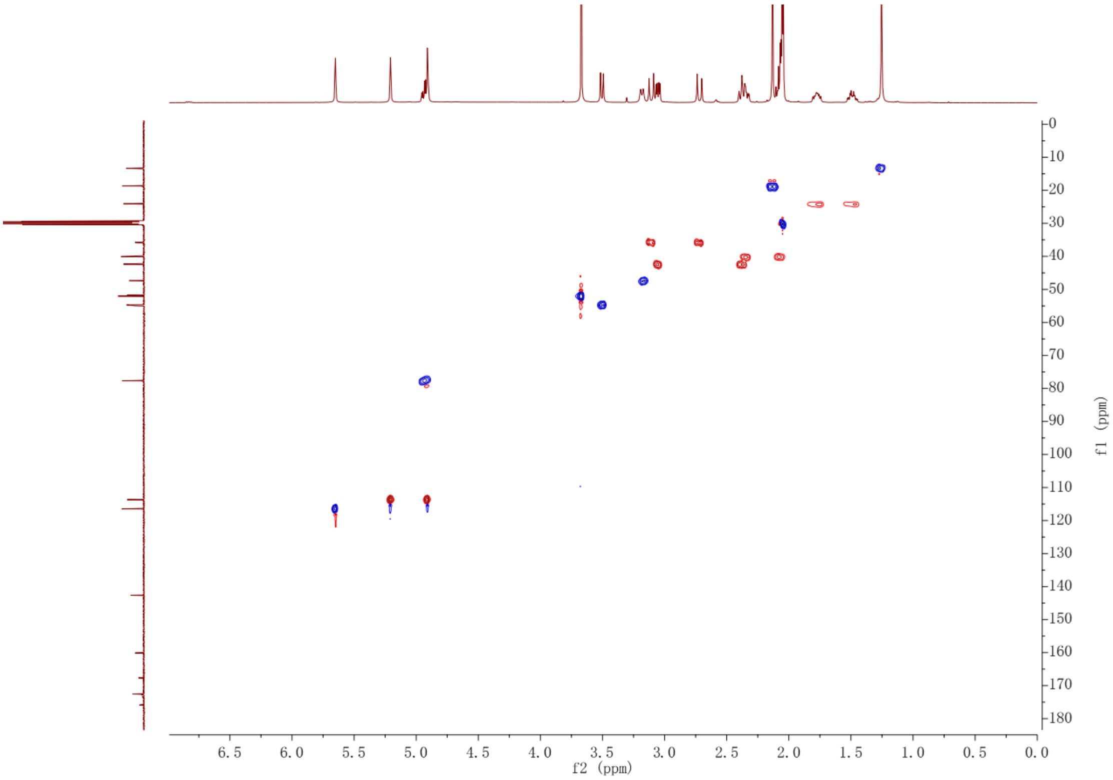


**Figure S10.** HSQC spectrum of hypoxyterpoid B (**2**; 500 MHz, Acetone-$d_6$)

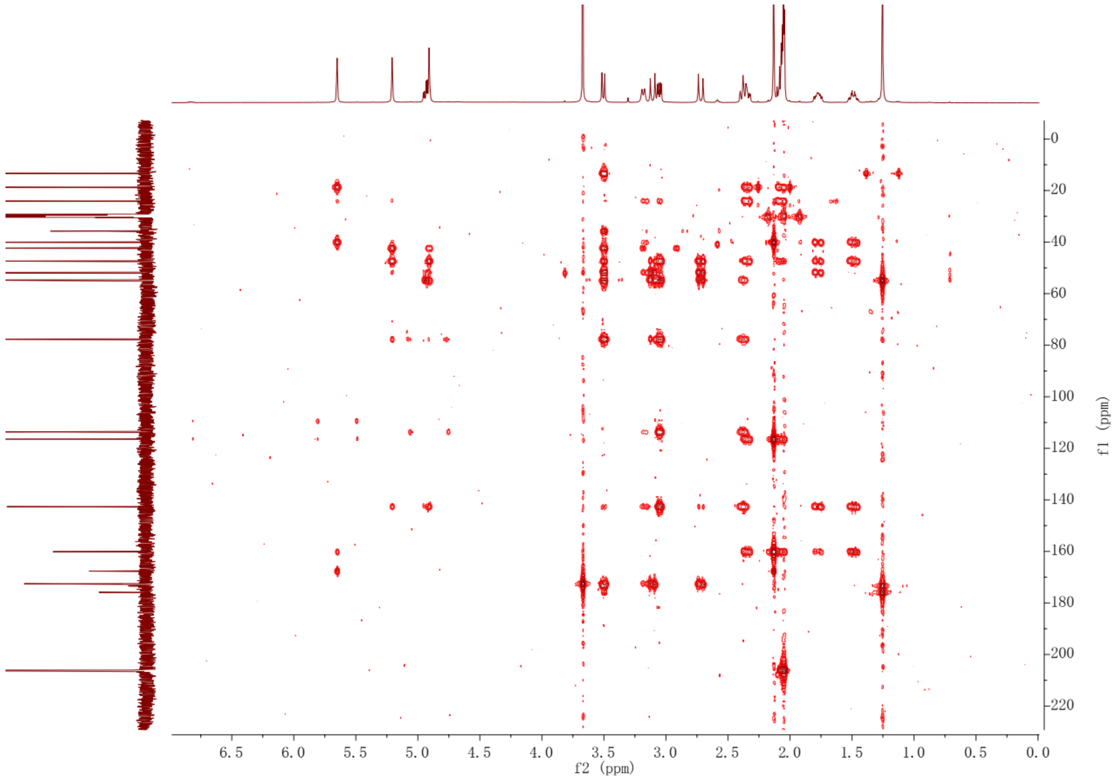


**Figure S11.** HMBC spectrum of hypoxyterpoid B (**2**; 500 MHz, Acetone-$d_6$)

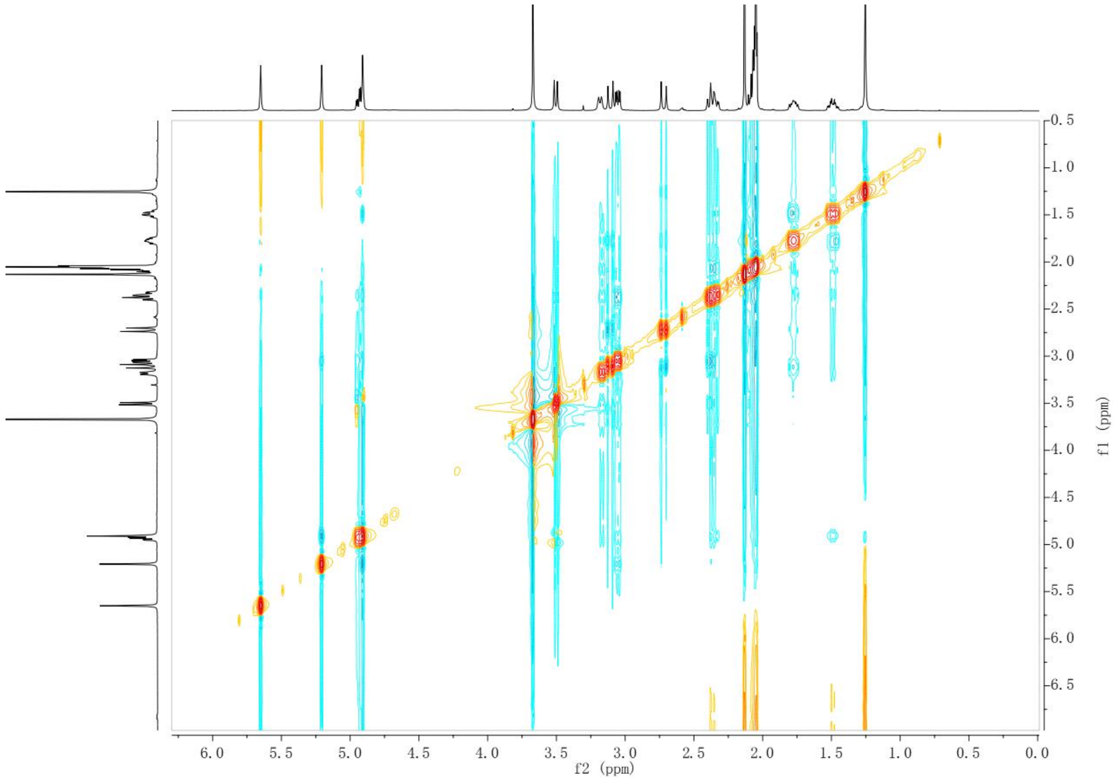


**Figure S12.** ROESY spectrum of hypoxyterpoid B (**2**; 500 MHz, Acetone-$d_6$)

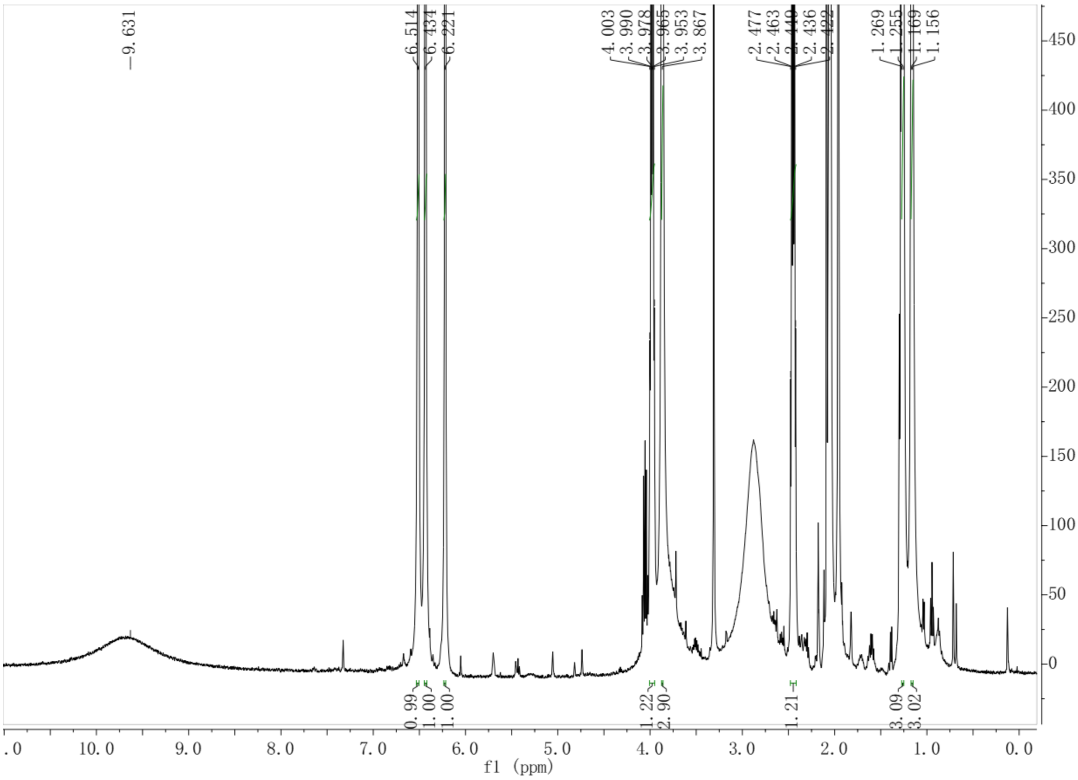


**Figure S13.** $^{1}$H NMR spectrum of hypoxymarin A (**4**; 500 MHz, Acetone-$d_6$)

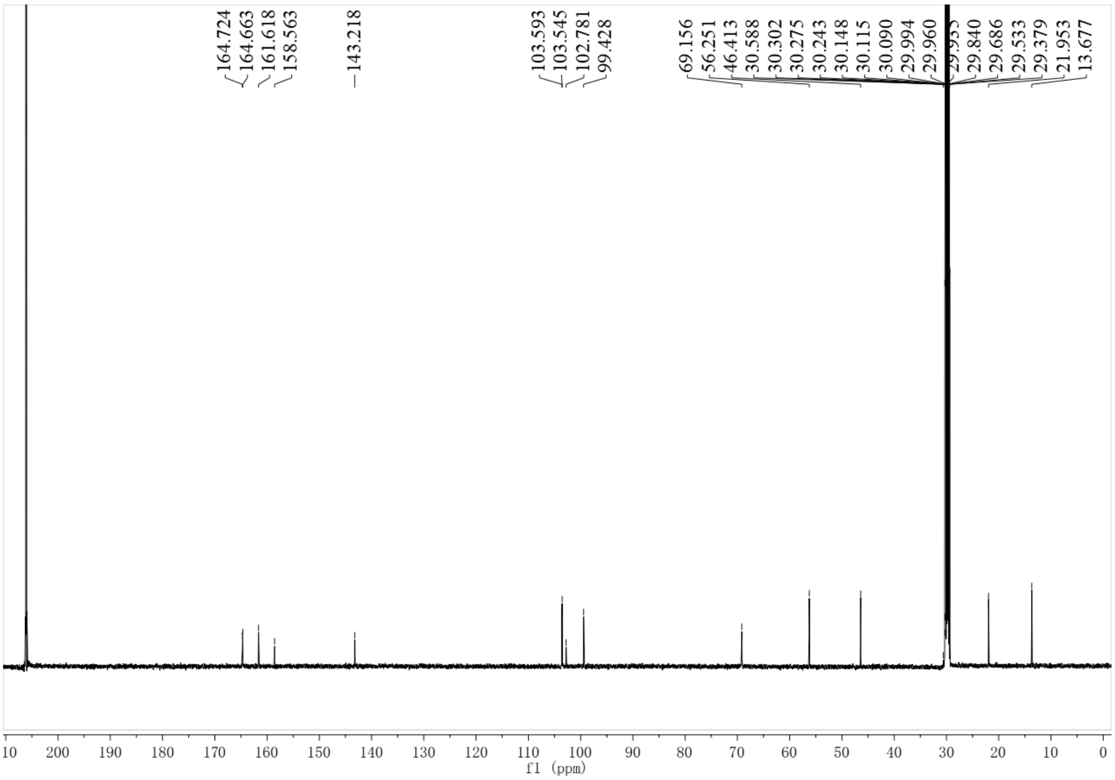


**Figure S14.** $^{13}$C NMR spectrum of hypoxymarin A (**4**; 125 MHz, Acetone-$d_6$)

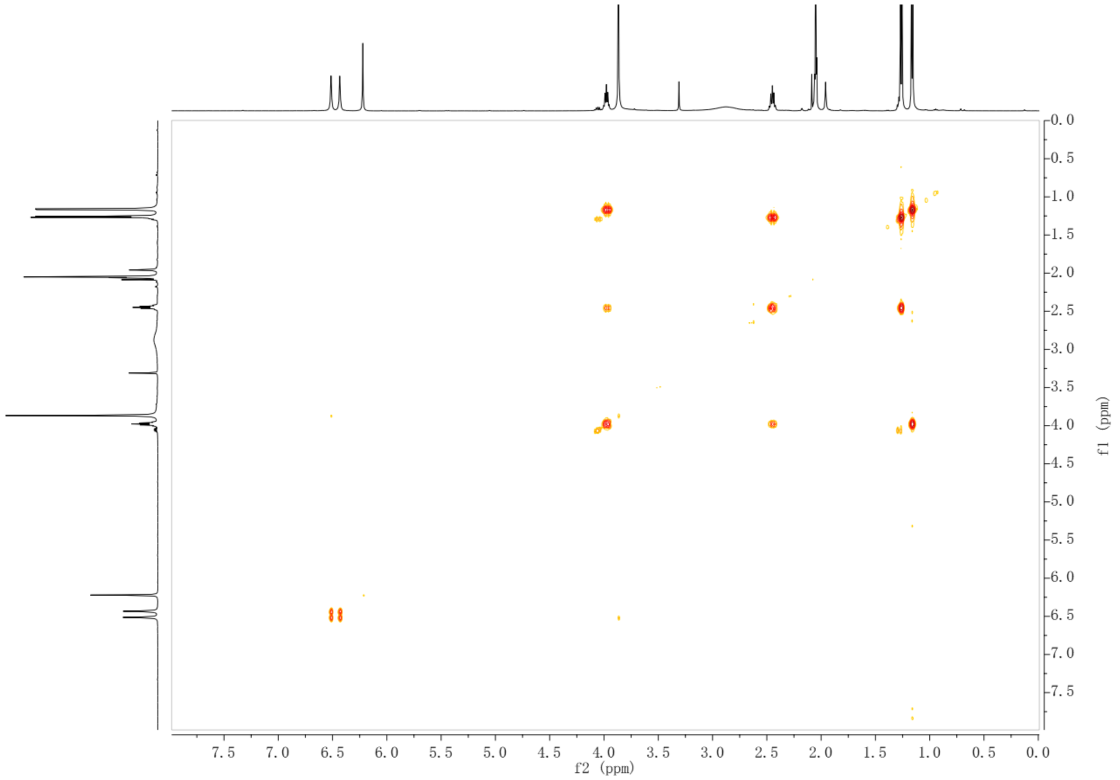


**Figure S15.** $^{1}H$-$^{1}H$ COSY spectrum of hypoxymarin A (**4**; 500 MHz, Acetone-$d_6$)

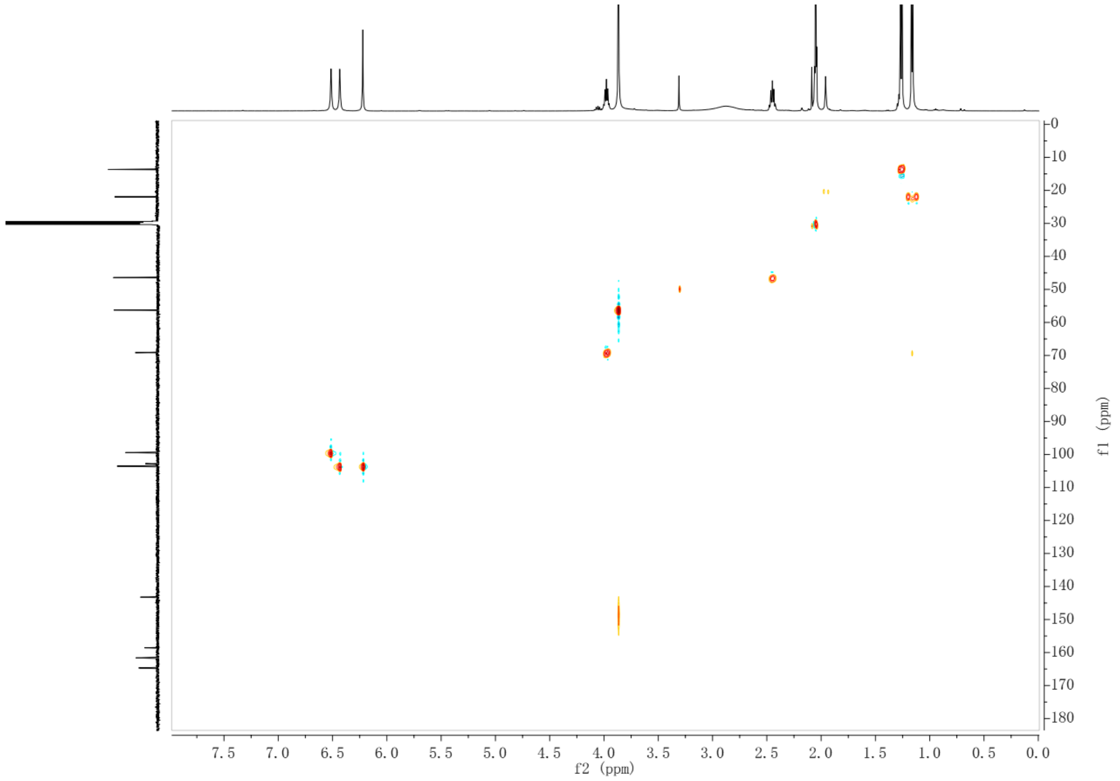


**Figure S16.** HSQC spectrum of hypoxymarin A (**4**; 500 MHz, Acetone-$d_6$)

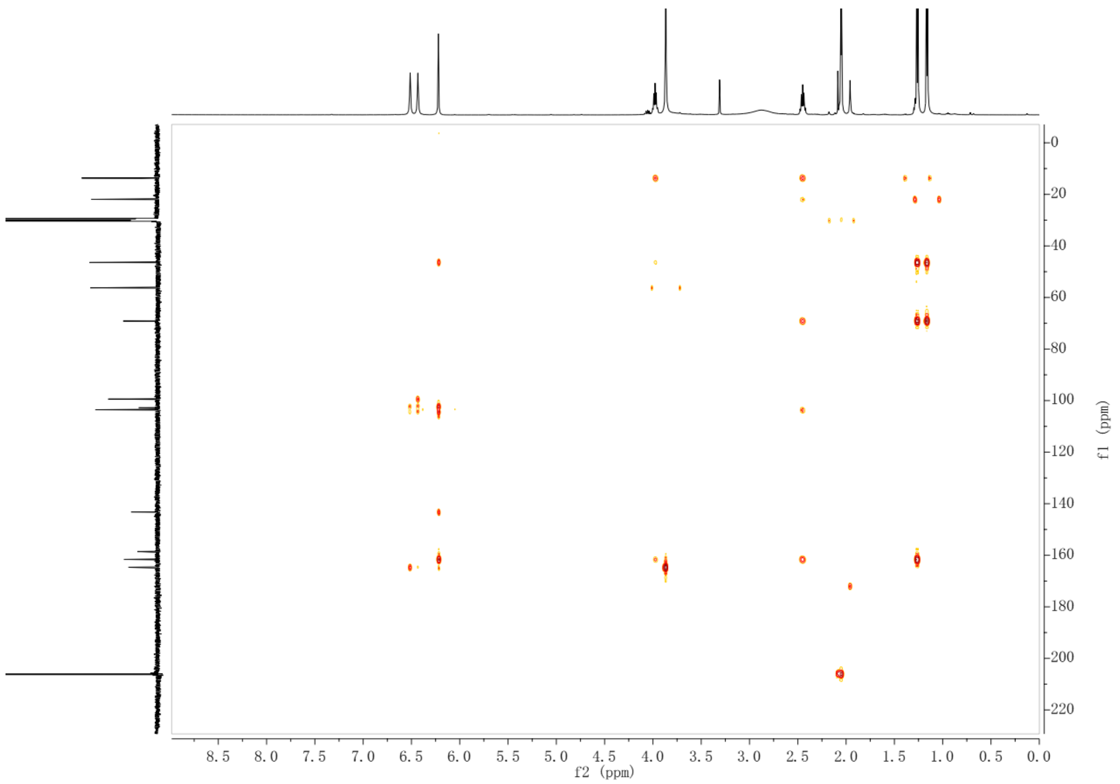


**Figure S17.** HMBC spectrum of hypoxymarin A (**4**; 500 MHz, Acetone-$d_6$)

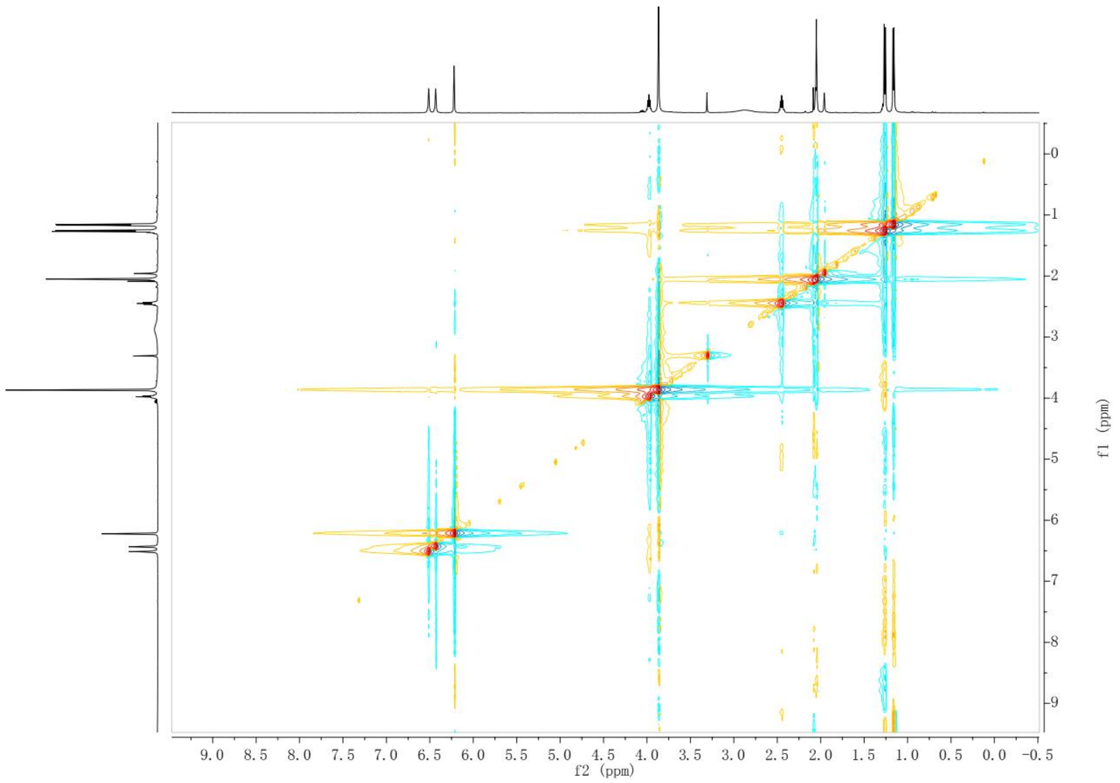


**Figure S18.** ROESY spectrum of hypoxymarin A (**4**; 500 MHz, Acetone-$d_6$)

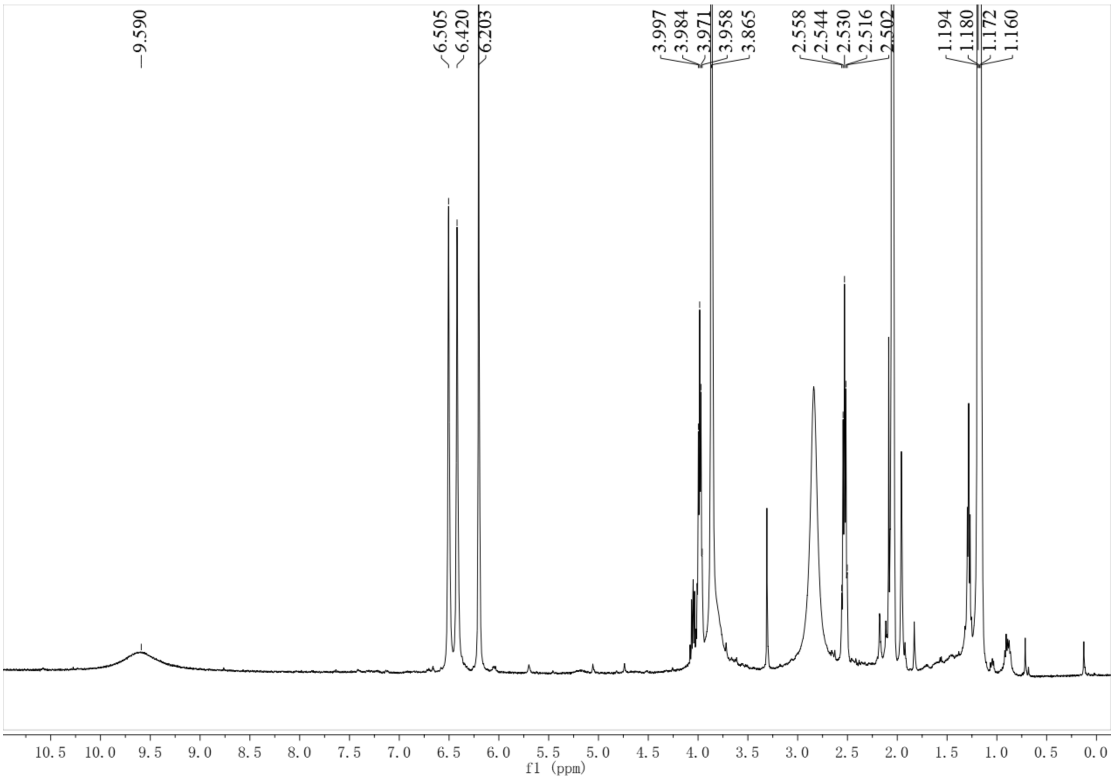


**Figure S19.** $^1$H NMR spectrum of hypoxymarin B (**5**; 500 MHz, Acetone-$d_6$)

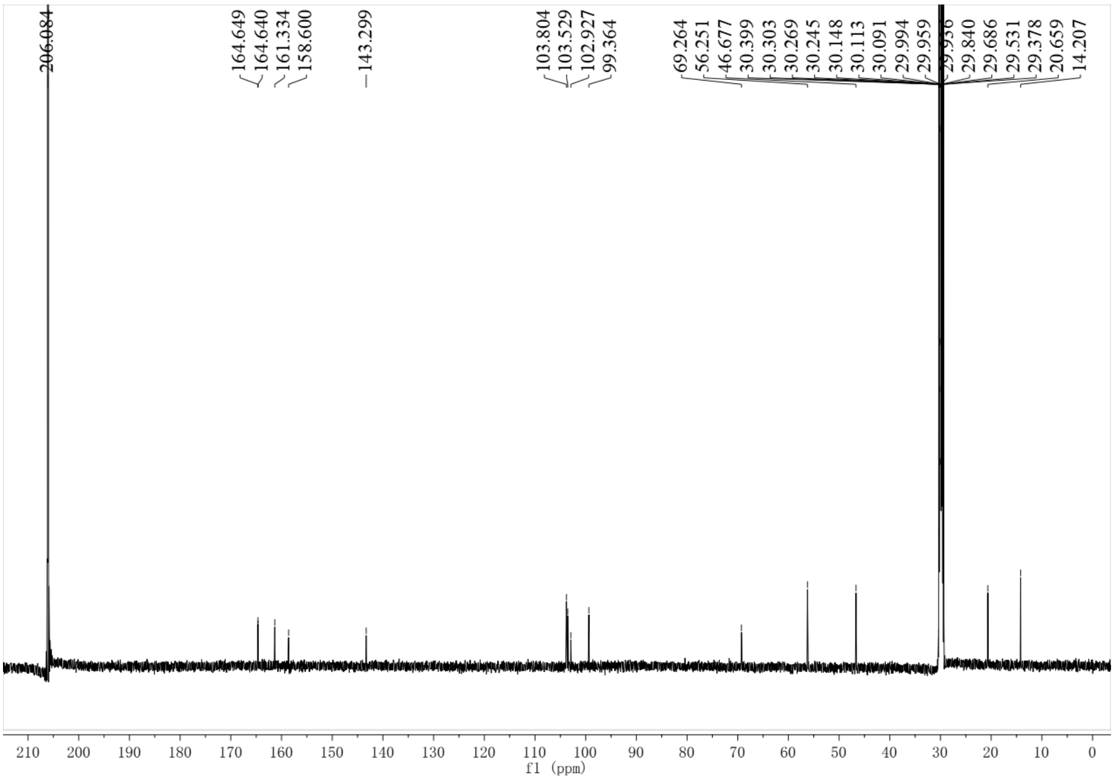


**Figure S20.** $^{13}$C NMR spectrum of hypoxymarin B (**5**; 125 MHz, Acetone-$d_6$)

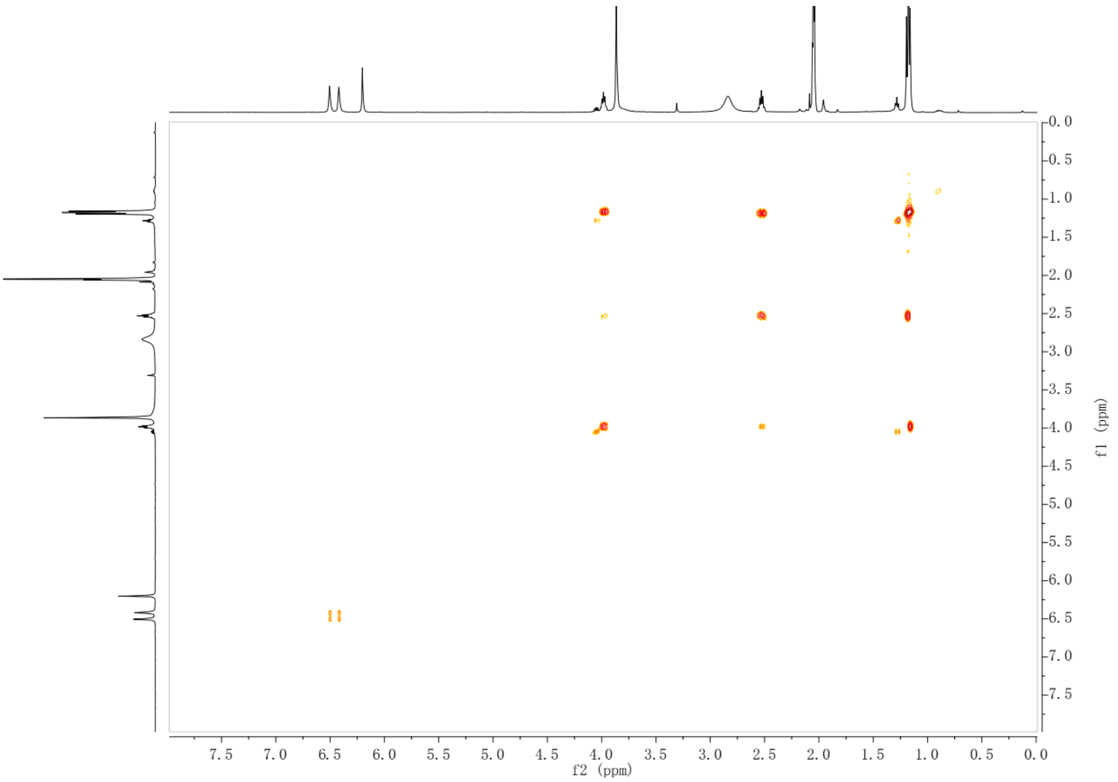


**Figure S21.** $^{1}$H-$^{1}$H COSY spectrum of hypoxymarin B (**5**; 500 MHz, Acetone-$d_6$)

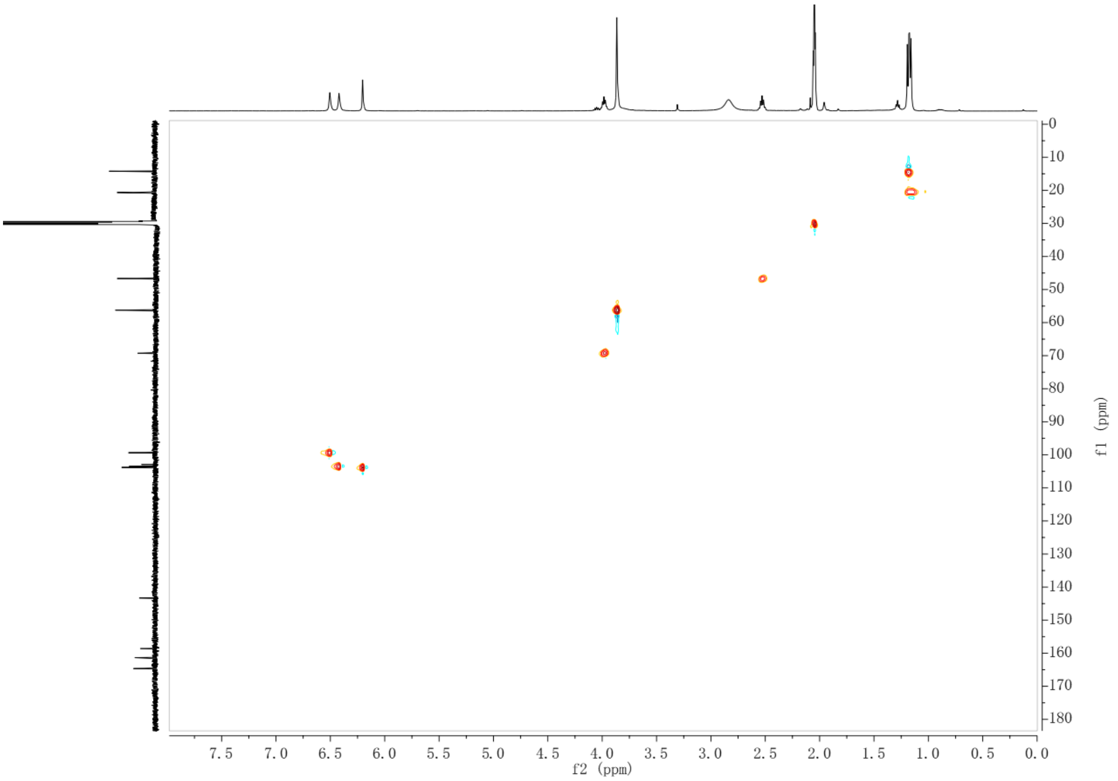


**Figure S22.** HSQC spectrum of hypoxymarin B (**5**; 500 MHz, Acetone-$d_6$)

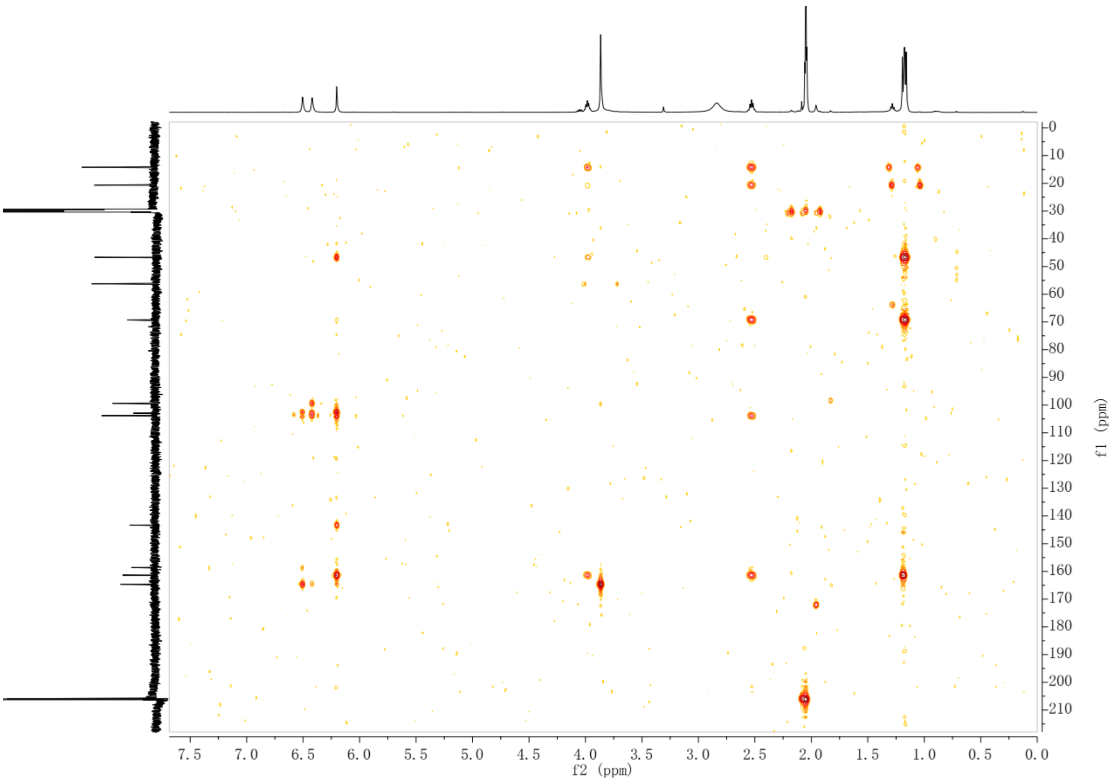


**Figure S23.** HMBC spectrum of hypoxymarin B (**5**; 500 MHz, Acetone-$d_6$)

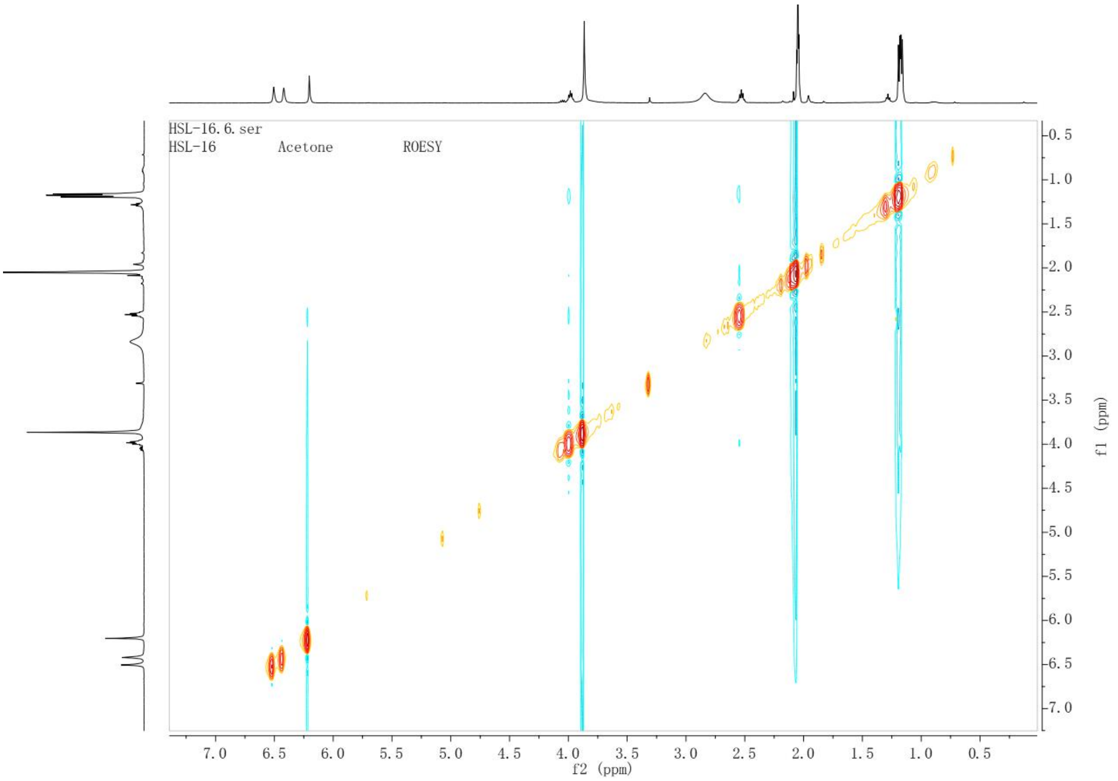


**Figure S24.** ROESY spectrum of hypoxymarin B (**5**; 500 MHz, Acetone-$d_6$)

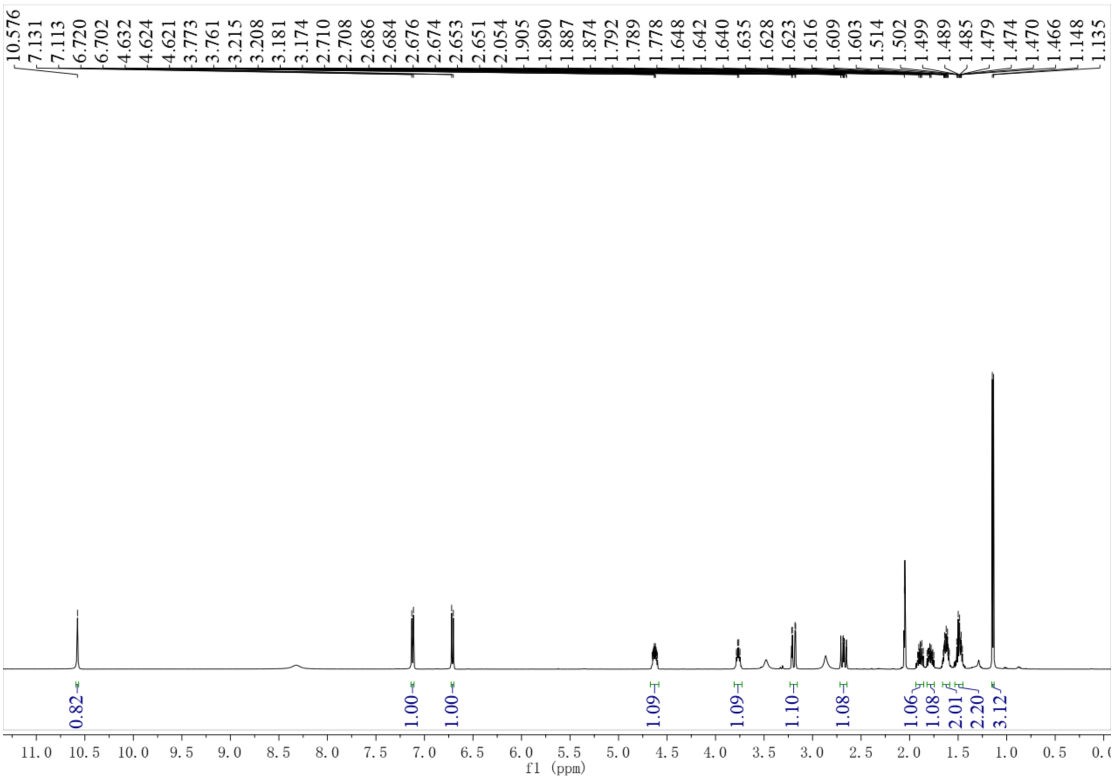


**Figure S25.** $^{1}$H NMR spectrum of hypoxymarin C (**6**; 500 MHz, Acetone-$d_6$)

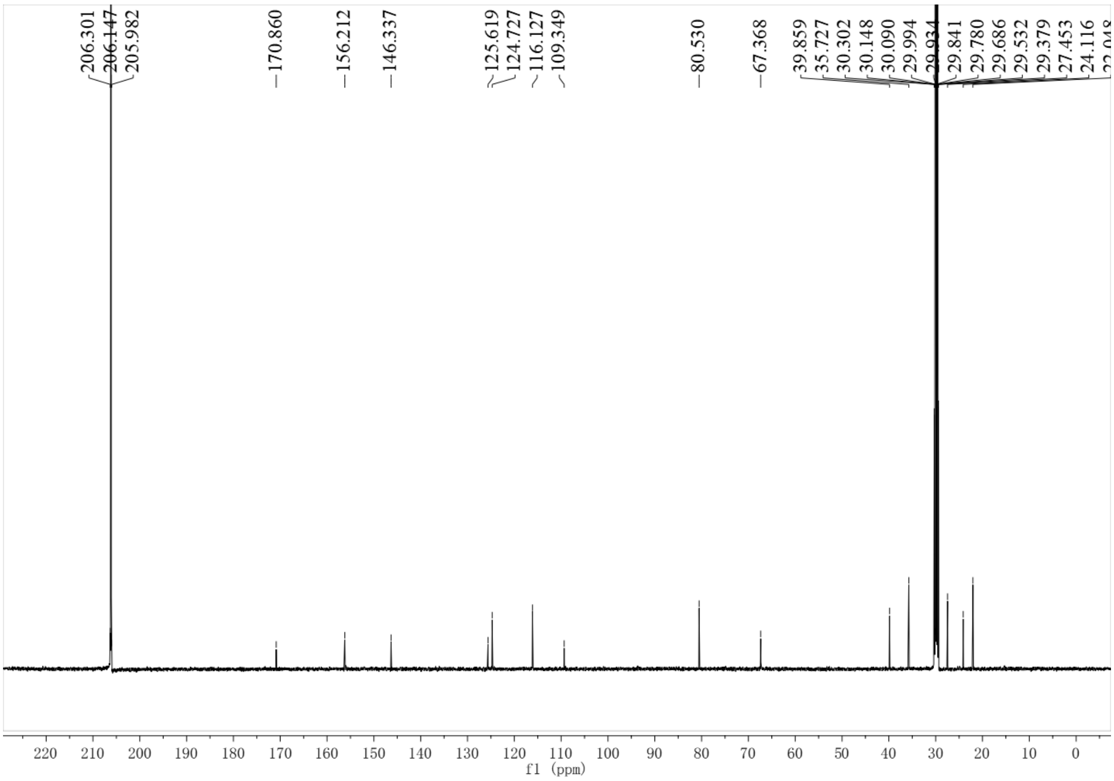


**Figure S26.** $^{13}$C NMR spectrum of hypoxymarin C (**6**; 125 MHz, Acetone-$d_6$)

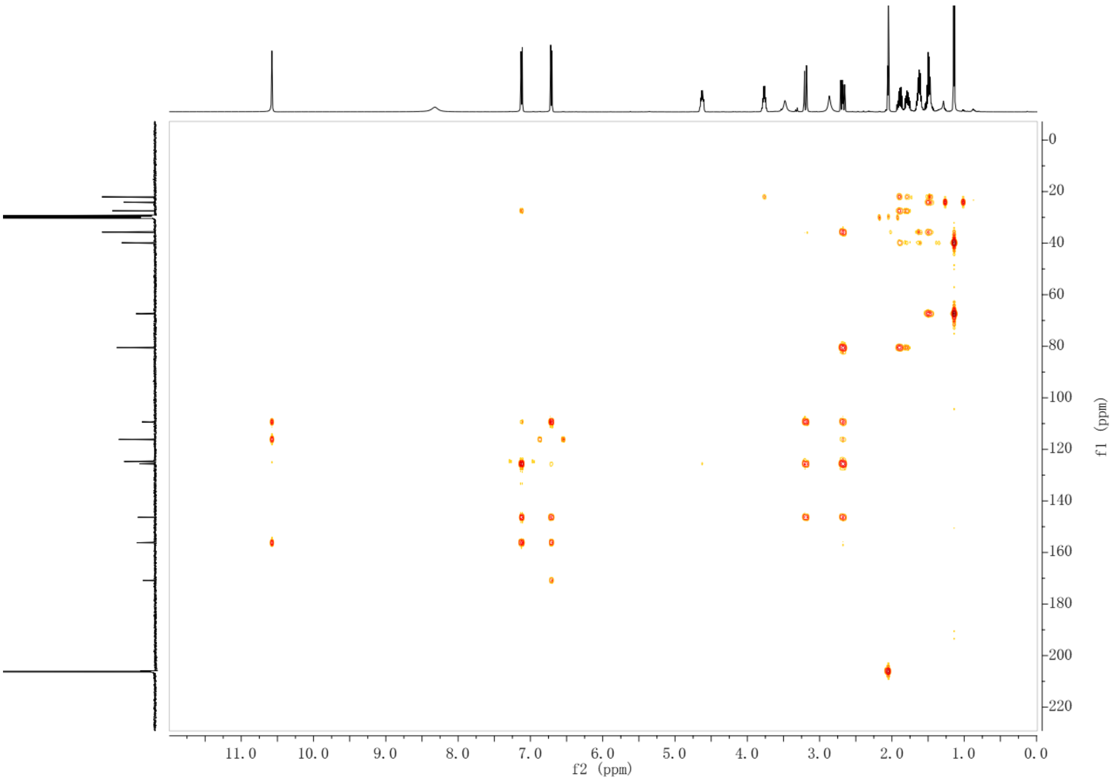


**Figure S27.** HMBC spectrum of hypoxymarin C (**6**; 500 MHz, Acetone-$d_6$)

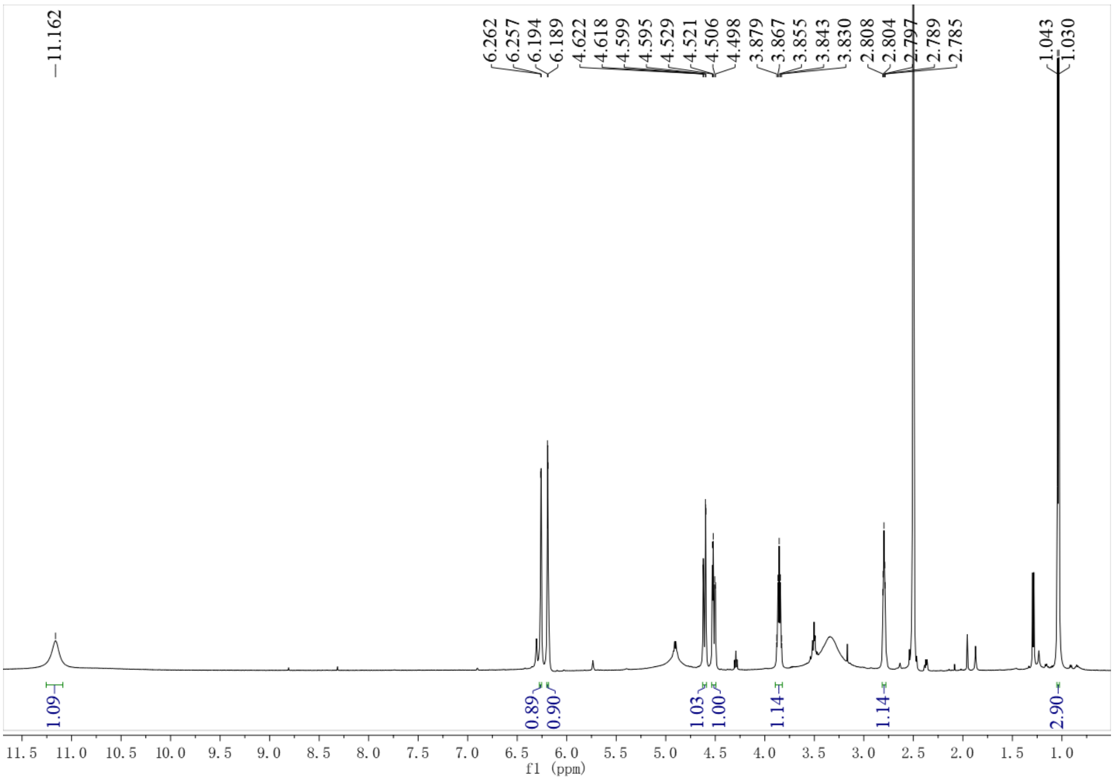


**Figure S28.** $^{1}$H NMR spectrum of hypoxymarin D (**7**; 500 MHz, DMSO-$d_6$)

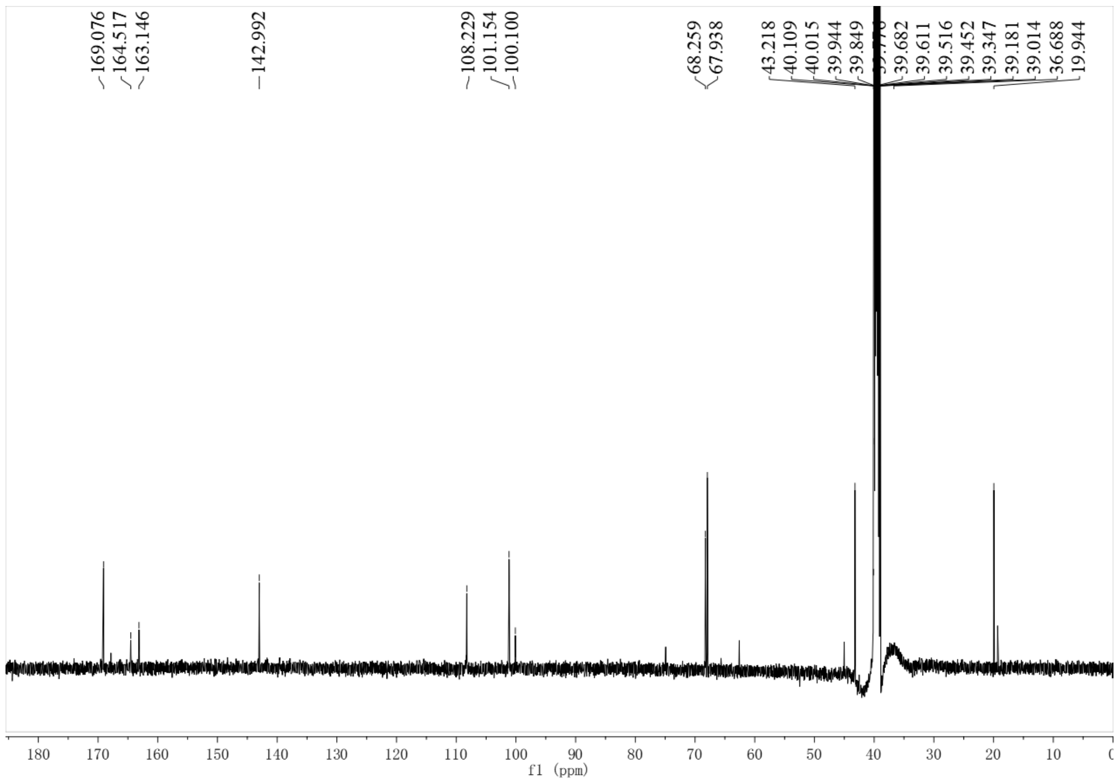


**Figure S29.** $^{13}C$ NMR spectrum of hypoxymarin D (**7**; 125 MHz, DMSO-$d_6$)

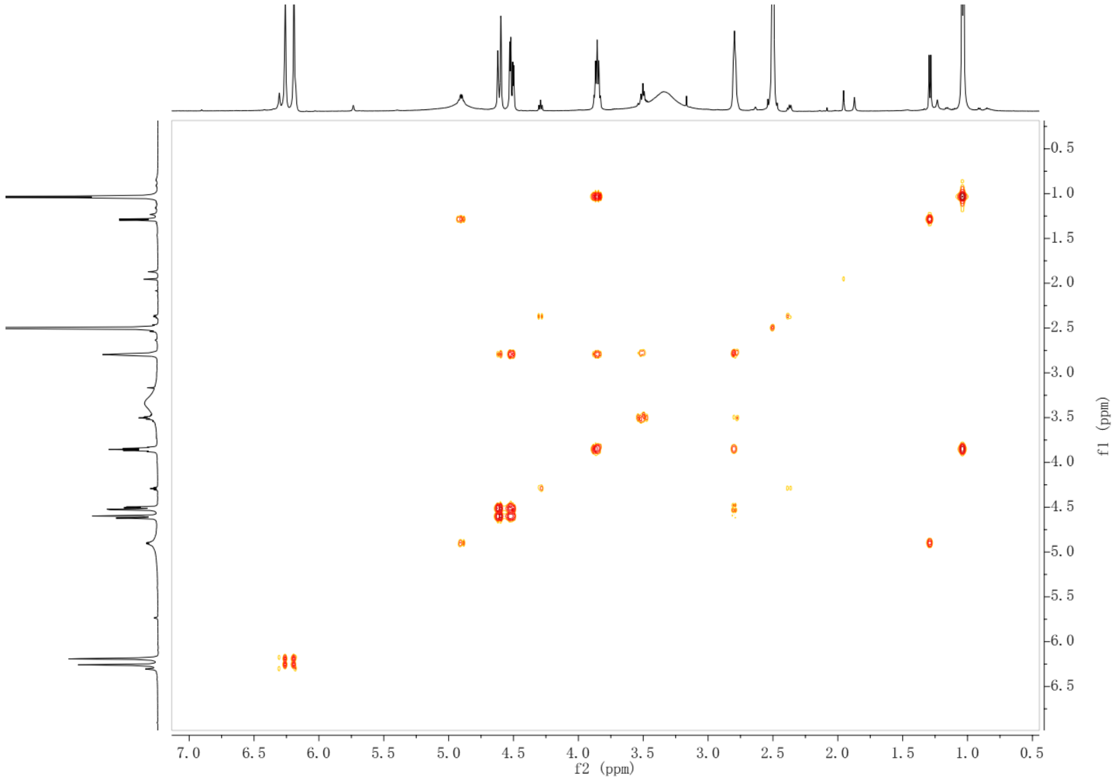


**Figure S30.** $^{1}H$-$^{1}H$ COSY spectrum of hypoxymarin D (**7**; 500 MHz, DMSO-$d_6$)

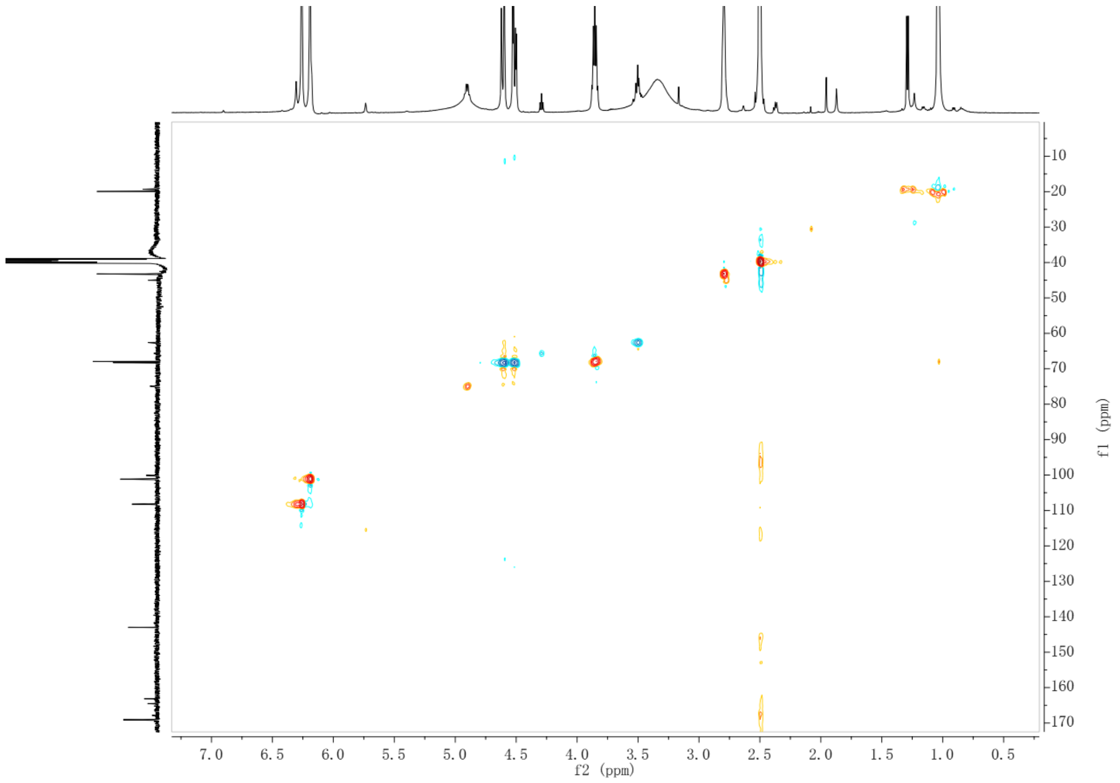


**Figure S31.** HSQC spectrum of hypoxymarin D (**7**; 500 MHz, DMSO-$d_6$)

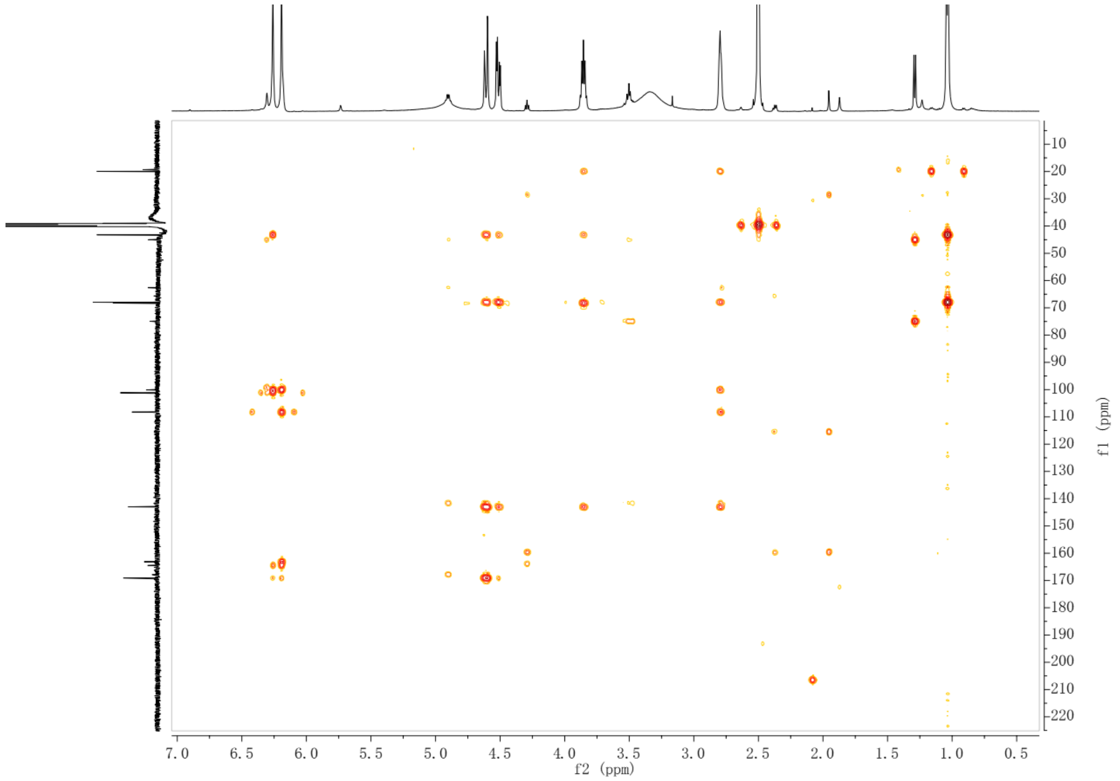


**Figure S32.** HMBC spectrum of hypoxymarin D (**7**; 500 MHz, DMSO-$d_6$)

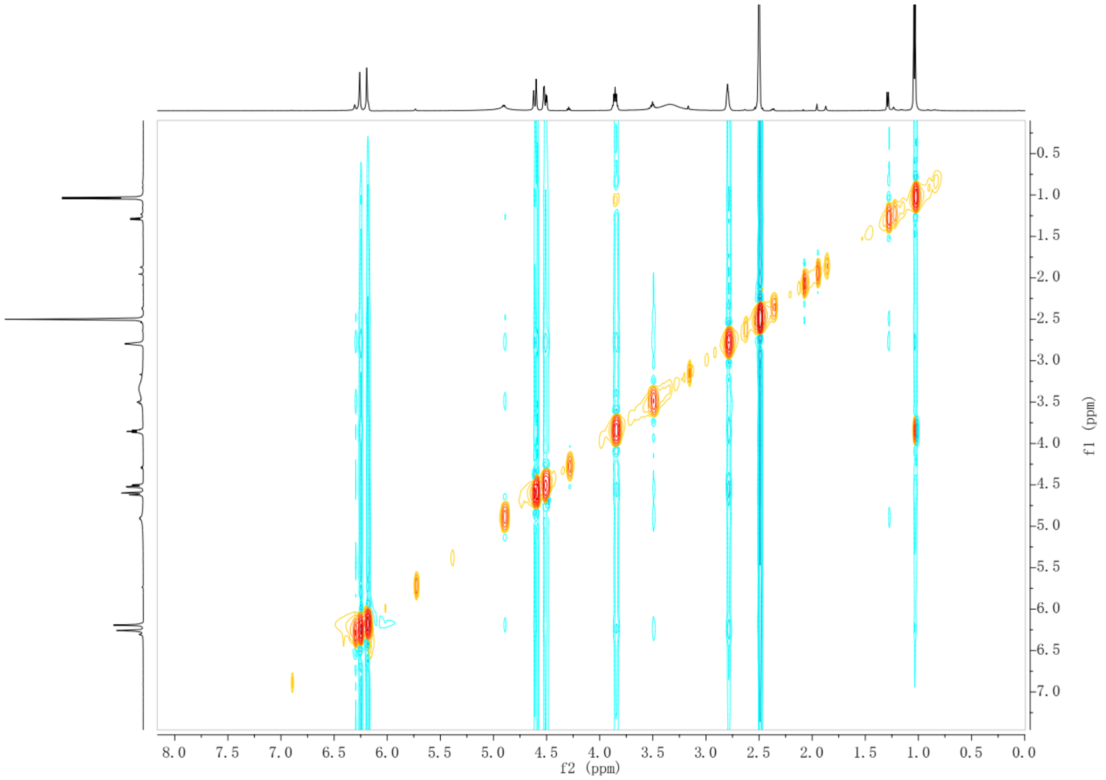


**Figure S33.** ROESY spectrum of hypoxymarin D (**7**; 500 MHz, DMSO-$d_6$)

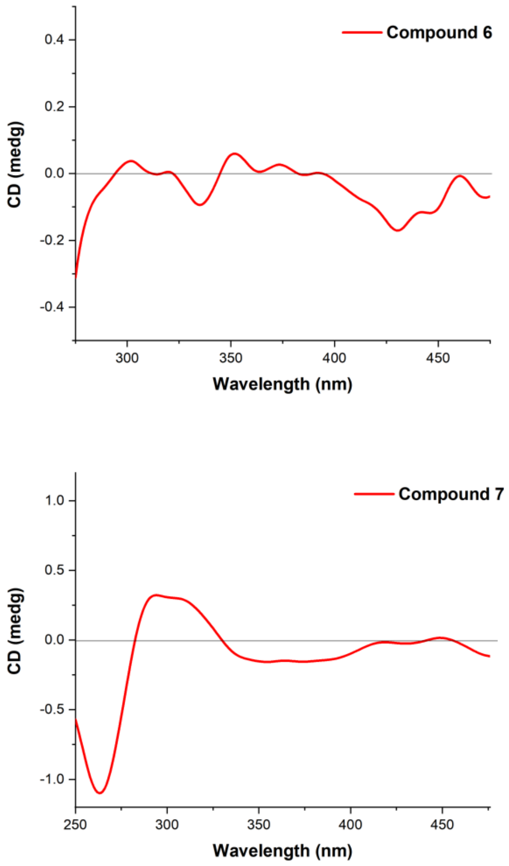


**Figure S34.** ECD spectra of the [$Rh_2(OCOCF_3)_4$] complexes of compounds **6** and **7** with the intrinsic ECD spectrum subtracted

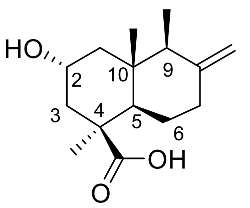


**1a**

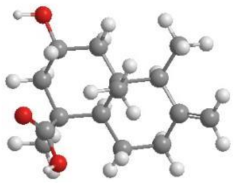

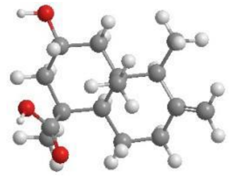

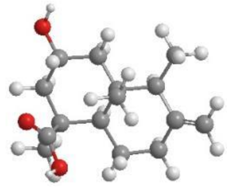

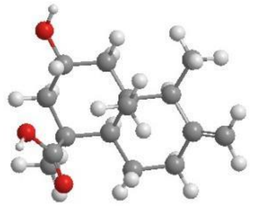

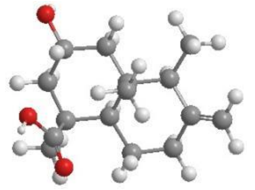

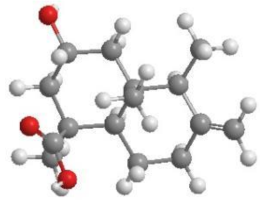

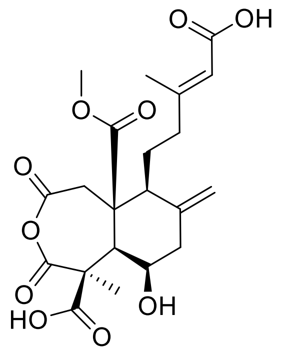


**2a**

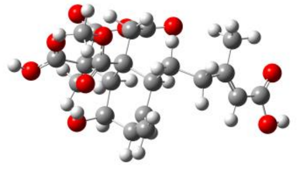

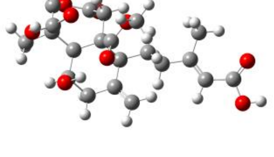

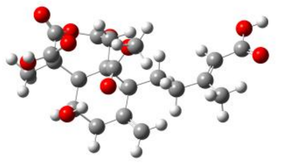

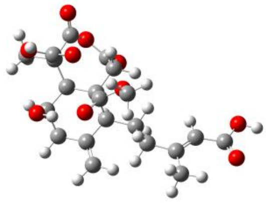

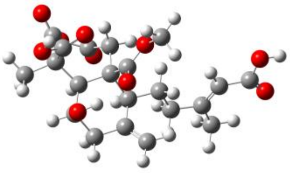

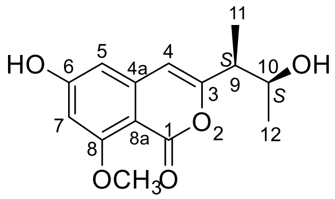


**4a**

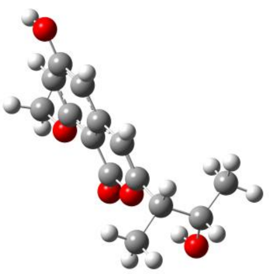

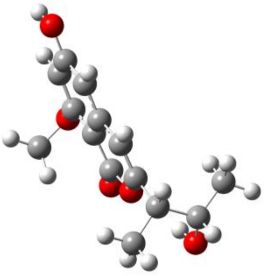

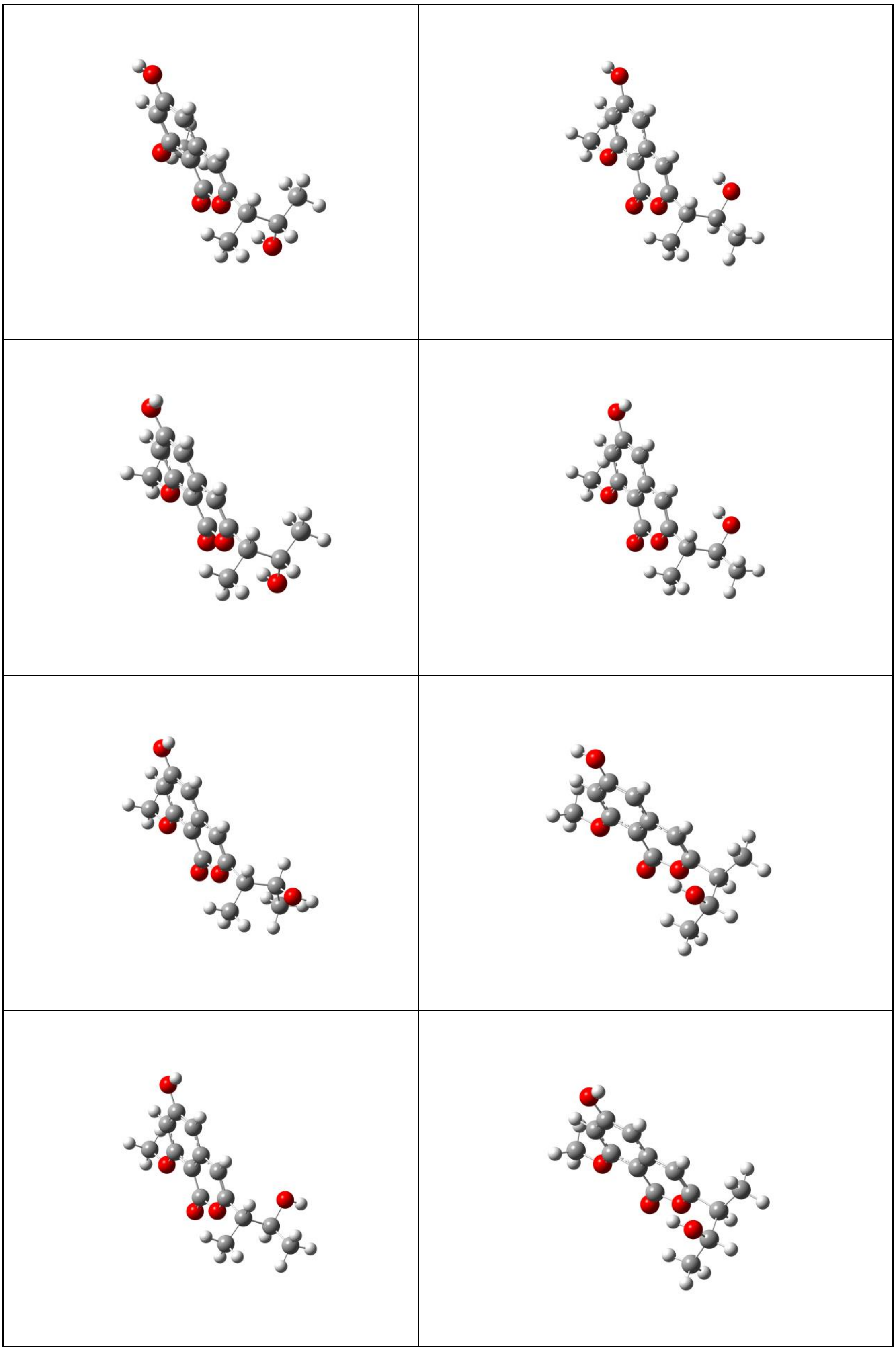

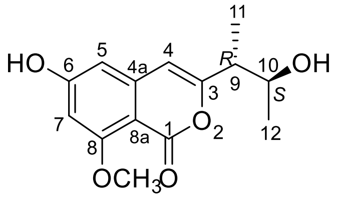


**4c**

| | |
|---|---|
| 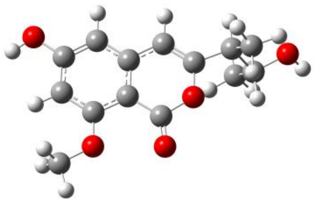 | 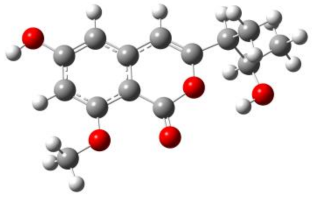 |
| 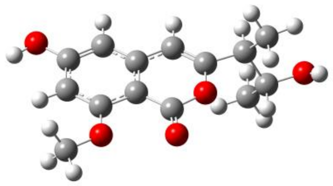 | 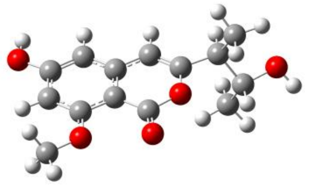 |
| 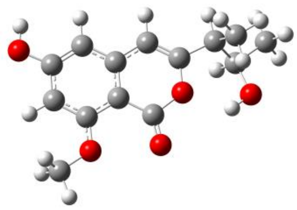 | 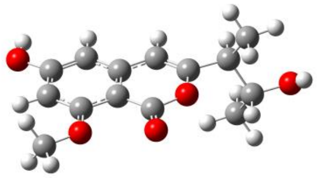 |

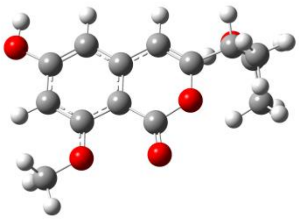

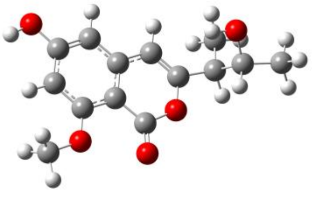

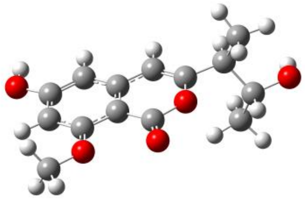

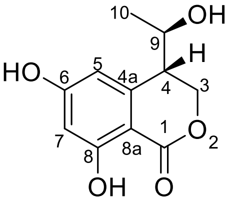


**7a**

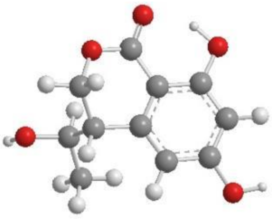

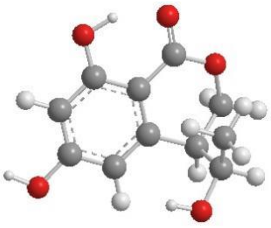

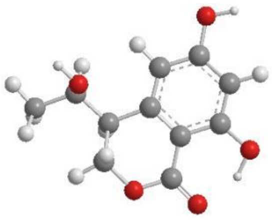

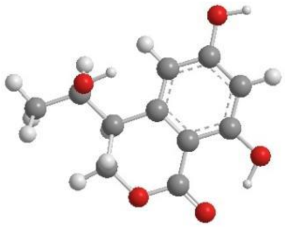

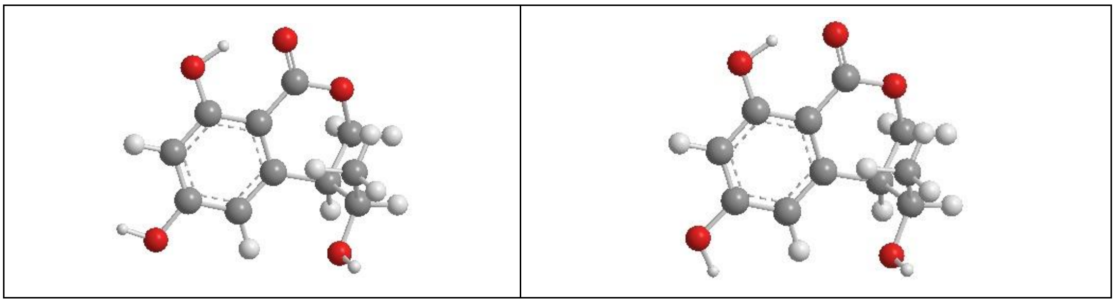

**Figure S35.** ECD conformers of **1**, **2, 4** and **7**
